# FoxO restricts growth and differentiation of cells with elevated TORC1 activity under nutrient restriction

**DOI:** 10.1371/journal.pgen.1007347

**Published:** 2018-04-20

**Authors:** Katarzyna Nowak, Avantika Gupta, Hugo Stocker

**Affiliations:** Institute of Molecular Systems Biology, ETH Zürich, Auguste-Piccard-Hof 1, Zürich, Switzerland; Harvard Medical School, Howard Hughes Medical Institute, UNITED STATES

## Abstract

TORC1, a central regulator of cell survival, growth, and metabolism, is activated in a variety of cancers. Loss of the tumor suppressors PTEN and Tsc1/2 results in hyperactivation of TORC1. Tumors caused by the loss of PTEN, but not Tsc1/2, are often malignant and have been shown to be insensitive to nutrient restriction (NR). In *Drosophila*, loss of PTEN or Tsc1 results in hypertrophic overgrowth of epithelial tissues under normal nutritional conditions, and an enhanced TORC1-dependent hyperplastic overgrowth of *PTEN* mutant tissue under NR. Here we demonstrate that epithelial cells lacking Tsc1 or Tsc2 also acquire a growth advantage under NR. The overgrowth correlates with high TORC1 activity, and activating TORC1 downstream of Tsc1 by overexpression of *Rheb* is sufficient to enhance tissue growth. In contrast to cells lacking PTEN, *Tsc1* mutant cells show decreased PKB activity, and the extent of *Tsc1* mutant overgrowth is dependent on the loss of PKB-mediated inhibition of the transcription factor FoxO. Removal of FoxO function from *Tsc1* mutant tissue induces massive hyperplasia, precocious differentiation, and morphological defects specifically under NR, demonstrating that FoxO activation is responsible for restricting overgrowth of *Tsc1* mutant tissue. The activation status of FoxO may thus explain why tumors caused by the loss of Tsc1–in contrast to PTEN–rarely become malignant.

## Author summary

Shortage of nutrients slows down cellular metabolism and protein synthesis, thereby restraining growth and proliferation of cells. Pre-cancerous cells deficient in specific tumor suppressors are resistant to these starvation-induced growth constraints, and instead gain a growth advantage over the surrounding tissue. We study the tumor suppressor genes *PTEN* and *Tsc1* in *Drosophila*. Loss of either PTEN or Tsc1 causes an overgrowth in epithelial tissues. We showed previously that the overgrowth of *PTEN* mutant cells is enhanced upon starvation due to an increase in proliferation. Here, we demonstrate that *Tsc1* mutant cells also acquire a growth advantage upon starvation, but this is primarily due to an increase in cell size. Genetic analyses revealed that this difference in the growth mechanisms of mutant cells exists because the transcription factor FoxO is activated in *Tsc1* mutant cells. Loss of FoxO function overcomes the proliferation brake in *Tsc1* mutant cells, resulting in massively overgrown tissues. In addition, tissues with low FoxO activity show signs of precocious differentiation. Thus, FoxO plays a key role in determining the fate of pre-cancerous cells.

## Introduction

In multicellular organisms, cellular and tissue growth is tightly coordinated with the local environment. Stress conditions like a lack of nutrients or growth factors prevent cell growth and proliferation [1]. In contrast, cancer cells are insensitive to anti-growth signals imposed by the environment and often grow better under stress conditions [2, 3].

It is well established that target of rapamycin complex 1 (TORC1) and phosphatidylinositol 3-kinase (PI3K) coordinate tissue growth with nutrient availability, and their deregulation can induce growth irrespective of the nutritional status [4]. Indeed, activation of these pathways due to the loss of their negative regulators, tuberous sclerosis complex 1 and 2 (Tsc1 and Tsc2) and phosphatase and tensin homolog (PTEN), respectively, can cause cancer [5]. Upon loss of *PTEN*, the levels of the membrane-bound second messenger phosphatidylinositol-3,4,5-tris-phosphate (PIP3) increase, which in turn recruits various effector proteins, most notably protein kinase B (PKB, also known as Akt). Once activated at the membrane, PKB promotes growth and survival by phosphorylation and inhibition of the forkhead transcription factor FoxO and of tuberin (Tsc2). The inhibitory phosphorylation of Tsc2, as well as the loss of either Tsc1 or Tsc2, disrupts the GTPase-activating function of Tsc1/2 towards the small GTPase Rheb, which increases GTP-bound Rheb levels and ultimately activates TORC1 pro-growth activity on the lysosomal membrane [6]. This leads to protein synthesis and ribosomal biogenesis, at least in part via phosphorylation of S6 kinase (S6K) and 4E-BP1. High TORC1 activity also results in lowered PKB activation through an S6K-dependent negative feedback loop [6, 7], and in activation of FoxO-mediated transcription of growth-inhibitory genes [8].

The PI3K/PKB/TORC1 signaling axis is conserved between humans and invertebrate model organisms such as *Drosophila melanogaster* [1, 9]. Genetic manipulations of *Drosophila* epithelial tissues with Flipase (Flp)/Flipase Recognition Target (FRT)—mediated mitotic recombination facilitate the analysis of loss-of-function mutations in tumor suppressor genes such as *PTEN* or *Tsc1/2* [10–12]. Clonal loss of *PTEN* or *Tsc1*/2 results in mild overgrowth of mutant tissue predominantly caused by enlarged cells [11, 13–17]. In line with previous reports about NR-insensitivity of tumors with constitutively active PI3K [2], NR severely enhances *PTEN* clonal overgrowth in a hyperplastic manner [18]. Not only are *PTEN* mutant cells resistant to starvation, they also gain a proliferative advantage over their wild-type neighbors and non-autonomously reduce the growth of the surrounding tissue [18]. The involvement of *Tsc1/2* mutations in conferring NR insensitivity remains elusive since different studies led to conflicting conclusions. Whereas some studies demonstrated that mammalian cells lacking Tsc1/2 are resistant to amino acid starvation, others revealed that cells lacking Tsc2 are highly sensitive to starvation and die upon amino acid removal [19–22]. Moreover, unlike tumors caused by mutations in *PTEN*, tumors lacking Tsc1/2 have attenuated PKB signaling and are mostly benign [5]. However, little is known about the underlying mechanism determining the progression to malignant tumors.

Here, we demonstrate that NR confers a growth advantage to mitotic clones lacking Tsc1. In contrast to *PTEN* mutant cells, *Tsc1* deficient cells overgrow in a hypertrophic manner under NR. Furthermore, whereas FoxO activity is suppressed in *PTEN* mutant cells due to high PKB activity, it is strongly activated in *Tsc1* mutant cells as a result of reduced PKB signaling and restricts hyperplastic overgrowth under NR. Combined removal of FoxO and Tsc1 under NR provokes a dramatic induction of hyperplasia, establishing the FoxO activation status as an important determinant of the extent of the overgrowth caused by the loss of Tsc1 under NR. Loss of FoxO also caused precocious differentiation in cells with high TORC1 activity under NR, unmasking a less studied function of FoxO in regulating differentiation of pre-cancerous cells.

## Results

### *Tsc1* mutant cells are resistant to starvation and gain a growth advantage under NR

**Fig 1:**
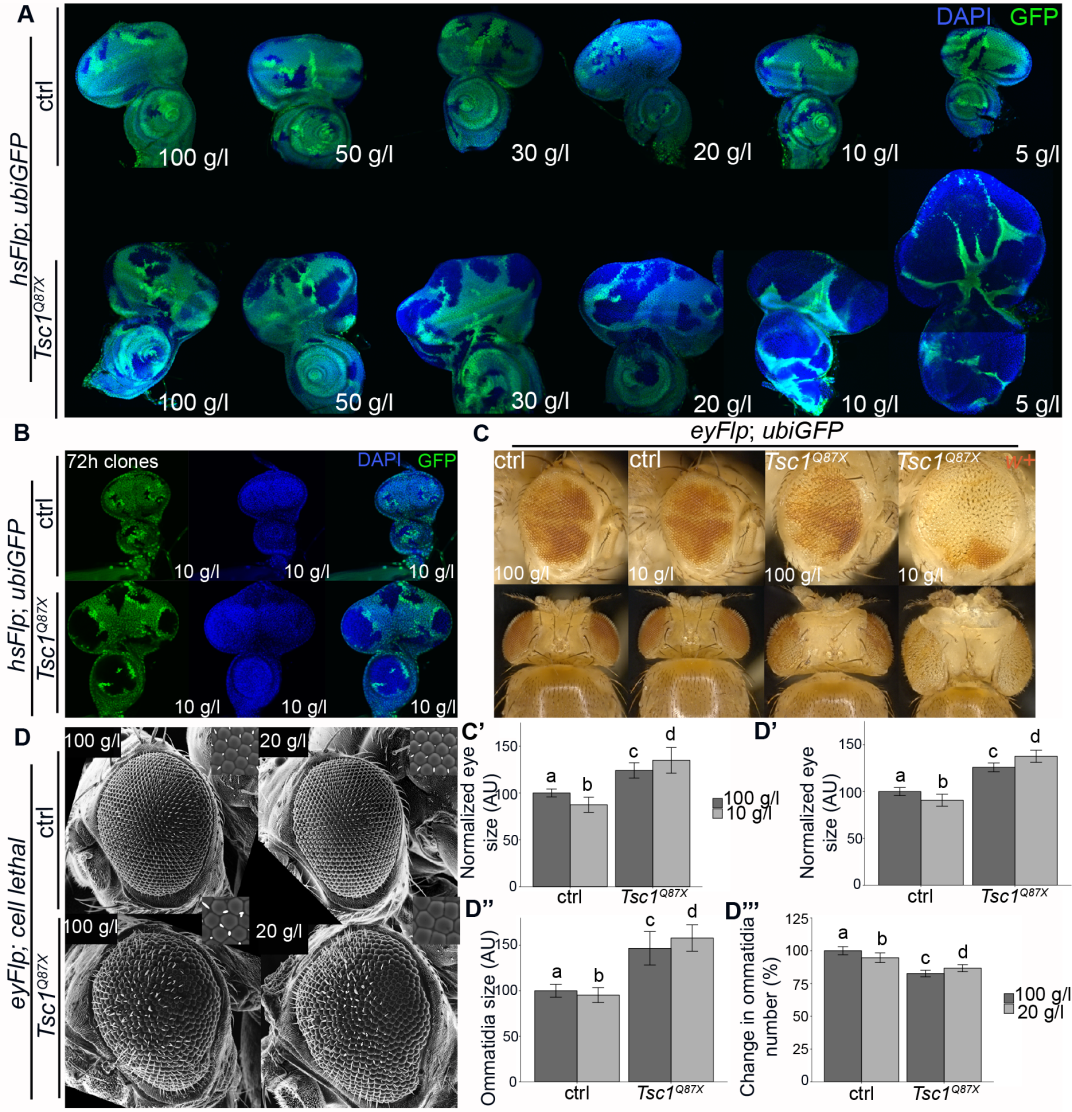
*Tsc1* mutant cells have a growth advantage under NR. **(A)** Third instar eye discs with hsFlp control or *Tsc1* mutant clones (marked by the absence of GFP) dissected from larvae reared under varying yeast concentrations. **(B)** Eye discs with hsFlp control or *Tsc1* mutant clones (marked by the absence of GFP) dissected 72 hours after clone induction from larvae reared on NR. **(C)** Eyes with eyFlp control or *Tsc1* mutant clones (marked by the absence of pigmentation) of animals reared on normal food and NR; **(C’)** quantification of eye sizes. **(D)** Scanning electron micrographs of eyes composed of control or *Tsc1* mutant tissue of animals reared on normal food and 20 g/l food, and the quantification of eye size **(D’)**, ommatidia size (n>260) **(D”)**, and ommatidia number **(D‴).**

To compare the effects of loss of Tsc1 with PTEN [18] on hyperactivation of TORC1 and cellular growth, we induced hsFlp/FRT-mediated clones of cells homozygous mutant for *Tsc1* in a heterozygous (and thus phenotypically normal) tissue. The autonomous growth properties of mutant cells and the non-autonomous interactions with the surrounding tissues were observed in eye-antennal imaginal discs dissected from larvae reared on food with varying yeast concentrations. The size of the discs bearing control clones correlated with the amount of yeast in the food (Fig 1A). In contrast, the discs with *Tsc1* mutant clones increased in size on food with reduced yeast concentrations due to enlarged mutant clones. This was accompanied by an increased growth disadvantage of the surrounding (heterozygous) tissue. For further analyses, we focused on 100 g/l yeast as normal food and 10 g/l yeast as NR. The *Tsc1* mutant clones consisted of larger and more cells, and they were already overgrown as compared to the adjacent wild-type twin spot 72 h after clone induction on NR (Fig 1B). Thus, the overgrowth was not due to the developmental delay caused by NR, but due to increased cell growth. We quantified the overgrowth of *Tsc1* mutant clones by measuring the size of adult eyes bearing eyFlp clones induced early in larval development. The eyes with control clones reduced in size upon NR (Fig 1C and 1C’). The eyes with *Tsc1* mutant clones were significantly larger than control on normal food, and the size was dramatically increased on NR. We also observed an almost complete absence of the neighboring tissue, similar to the situation in imaginal discs (Fig 1A). The size increase of *Tsc1* mutant heads upon NR was accompanied by a non-autonomous reduction in body size (shoulder area, Fig 1C and S1 Datasets), akin to the systemic effects observed upon eye-specific loss of *PTEN* [18].

Since Tsc1 functions in a complex with Tsc2, we analyzed whether loss of Tsc2 also confers a growth advantage to cells upon NR. We knocked down *Tsc2* specifically in the eye-antennal imaginal discs using the eyFlp-out system and quantified the size of adult eyes and eye discs. Knockdown of *Tsc2* caused an increase in the imaginal discs and adult eye sizes on normal food (S1 Fig). This overgrowth was massively exacerbated upon NR, causing compromised survival of larvae in the late third instar. Thus, an impaired function of either component of the tuberous sclerosis complex confers a growth advantage to cells under NR.

To determine how NR affects size and number of *Tsc1* mutant cells, we generated eyes that were composed almost completely of mutant tissue using the eyFlp cell lethal system (Fig 1D) [23]. Because the *Tsc1* mutant flies did not survive on 10 g/l yeast, we used 20 g/l yeast as NR condition. Since the loss of Tsc1 does not alter the composition of ommatidia (S2 Fig), we used ommatidia size and number as measures for cell size and number. Eyes mutant for *Tsc1* showed an increase in ommatidia size (Fig 1D”) and a decrease in ommatidia number (Fig 1D‴), indicating that the increase in total eye size (Fig 1D’) is due to larger ommatidia. The ommatidia number in *Tsc1* mutant eyes significantly increased on 20 g/l yeast food as compared to normal food, but there was a strong reduction as compared to control eyes on starvation. This suggests that the enhancement of overgrowth of *Tsc1* mutant eyes on NR is due to an enlargement of ommatidia as compared to normal food. We conclude that *Tsc1* mutant tissue overgrows in a hypertrophic manner, and this hypertrophic overgrowth is enhanced upon NR.

### Proliferating *Tsc1* mutant cells are susceptible to apoptosis

**Fig 2:**
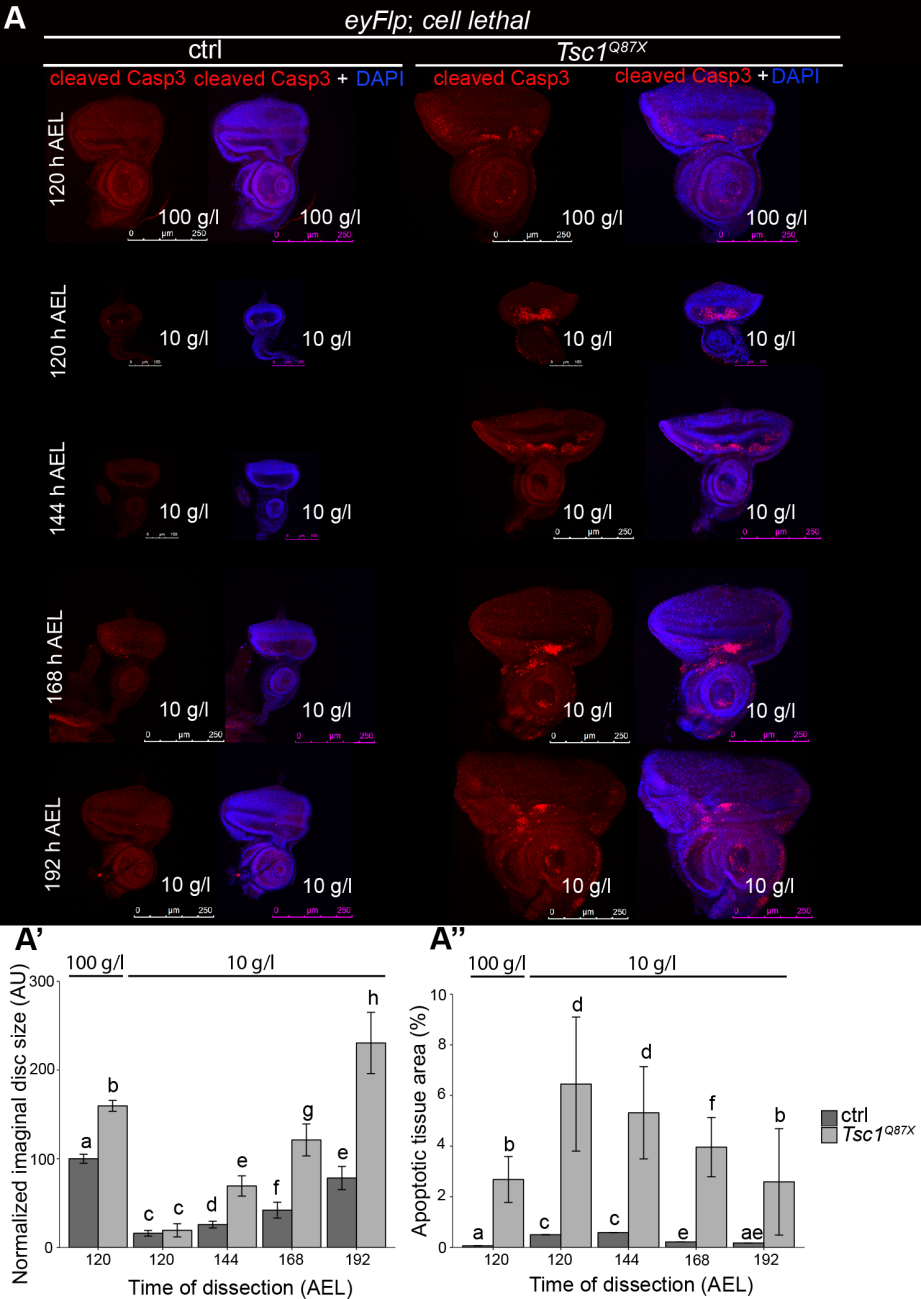
*Tsc1* mutant proliferating cells undergo massive apoptosis. **(A)** Cleaved Caspase-3 antibody staining (in red) of control or *Tsc1* mutant eye discs dissected at the indicated developmental points from larvae reared on normal food and NR; quantification of eye discs size **(A’)**, and percentage of apoptotic tissue area with respect to the total area of discs **(A”)**. Scale bars are 250 μm and 100 μm, respectively.

Analysis of the Tsc1 loss-of-function phenotype at different developmental stages under NR revealed that *Tsc1* mutant cells initially overproliferate in eye imaginal discs (Fig 1A and 1B) but yield a net hypertrophic overgrowth in the adult eyes (Fig 1D’ and 1D”). To test whether this phenotypic discrepancy is caused by apoptotic events, we immunostained discs mutant for *Tsc1* with antibodies directed against cleaved Caspase-3 at different developmental time points (Fig 2). Compared to control discs, *Tsc1* mutant discs displayed considerable levels of apoptosis on normal food. On NR, the amount of apoptotic tissue was increased anterior to the morphogenetic furrow, the region characterized by asynchronous mitoses. Thus, apoptotic elimination of *Tsc1* mutant cells at early developmental stages could account for the net hypertrophic overgrowth of *Tsc1* mutant tissue later in development. Consistent with our hypothesis, blocking cell death specifically in *Tsc1* mutant cells by expression of the anti-apoptotic baculovirus protein p35 enhanced the extent of the overgrowth under NR (S3A Fig).

Next, we investigated why the wild-type cells neighboring *Tsc1* mutant tissue suffer from an increased growth disadvantage under NR (Fig 1A, 1B and 1C). TORC1 promotes growth by regulating ribosomal biogenesis [6, 24]. We hypothesized that high TORC1-dependent pro-growth activity in *Tsc1* mutant tissues (see below) under NR could be the underlying cause for Minute-like outcompetition [25], where suboptimal slow dividing cells (Minute, losers) are removed by apoptosis from the local environment of fitter cells (Minute+, winners). To test this hypothesis, we performed cleaved Caspase-3 immunostaining on mosaic discs bearing hsFlp *Tsc1* clones (S3B Fig). Whereas apoptosis was consistently induced in *Tsc1* mutant cells, we only observed few dying neighboring cells, which is unlikely to account for the elimination of the surrounding (heterozygous and wild-type) tissue from the eye discs. Therefore, cell competition does not cause the overgrowth of *Tsc1* clones.

### Overgrowth of *Tsc1* mutant tissue requires TORC1 signaling

**Fig 3:**
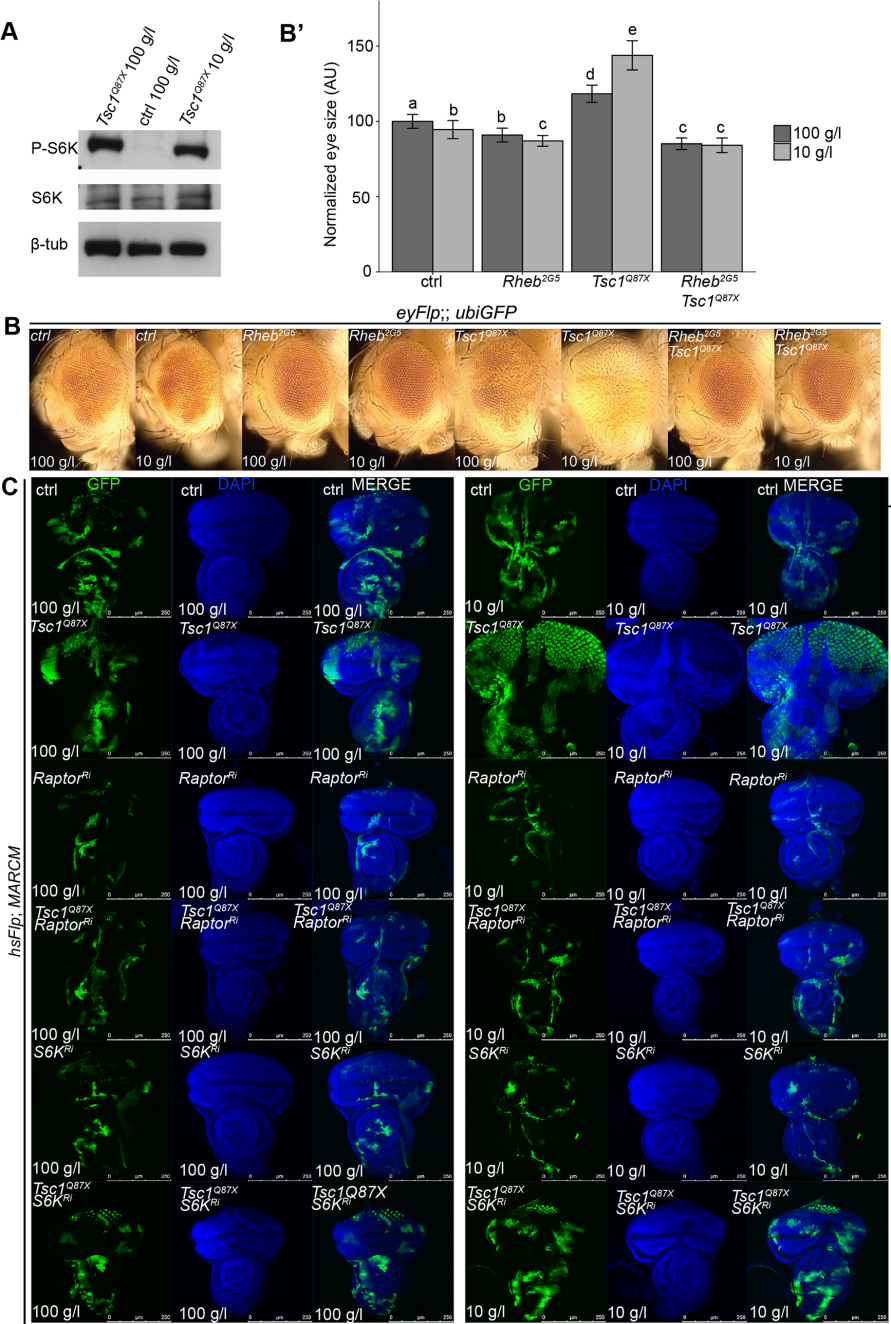
Overgrowth caused by loss of *Tsc1* is dependent on TORC1 signaling. **(A)** Western blot analysis of proteins extracted from the control or *Tsc1* mutant eye imaginal discs. The extremely small size of the control eye discs from animals reared on NR precluded the Western analysis. Total levels of S6K were unaltered. β**-**tubulin served as loading control. **(B)** Eyes with eyFlp control, *Tsc1*, *Rheb*, and combination of *Tsc1* and *Rheb* clones (marked by the absence of pigmentation) of animals reared under normal food and NR; **(B’)** quantification of eye size. **(C)** Eye discs bearing MARCM control and *Tsc1* mutant clones, with or without the knockdown of *Raptor* or *S6K* (marked by GFP) dissected from larvae reared on normal food or NR. Scale bars are 250 μm.

To examine the activation of TORC1 signaling in *Tsc1* mutant tissue, we monitored the phosphorylation of S6K (phospho-S6K) by Western blot analysis. Whereas the levels of phospho-S6K were low in wild-type discs, S6K phosphorylation was strongly induced in *Tsc1* mutant tissue and remained equally strong under NR (Fig 3A). Thus, TORC1 activity is not reduced by NR in *Tsc1* mutant tissue.

To test the requirement of TORC1 signaling for overgrowth of *Tsc1* mutant tissue on NR, we selectively inhibited its activity in *Tsc1* mutant clones by removing its activator Rheb [6, 21], by knocking down the TORC1 component Raptor [6] and the TORC1 effector S6K, and by overexpressing 4E-BP as well as a phospho-mutant form of 4E-BP that cannot be inactivated by TORC1-mediated phosphorylation (4E-BP^AA^) [26]. Removing Rheb or reducing Raptor and S6K function suppressed the *Tsc1* mutant overgrowth under normal conditions and NR (Fig 3B, 3B’ and 3C). In contrast, overexpression of either form of 4E-BP did not reduce *Tsc1* clonal overgrowth (S4 Fig), indicating that TORC1-mediated inhibition of 4E-BP is not required for the overgrowth phenotype in mitotic tissues.

We then asked whether activating TORC1 downstream of the tuberous sclerosis complex could promote tissue overgrowth under NR. Similar to *Tsc1* mutant clones, clones with overexpression of *Rheb* overgrew on NR (S5A Fig). Surprisingly, overexpression of *Rheb* using the eyFlp-out system led to smaller eyes under both food conditions (S5B and S5B’ Fig). Cleaved Caspase-3 immunostaining revealed that *Rheb*-expressing proliferating cells undergo massive apoptosis upon NR (S5C Fig). Thus, activation of TORC1 downstream of Tsc1/2 by expressing *Rheb* is sufficient to confer an initial growth advantage to cells under NR, but elevated levels appear to be detrimental, resulting in increased apoptosis, and ultimately less overgrown organs.

### PKB and FoxO are differentially activated in *PTEN* and *Tsc1* mutant tissues

**Fig 4:**
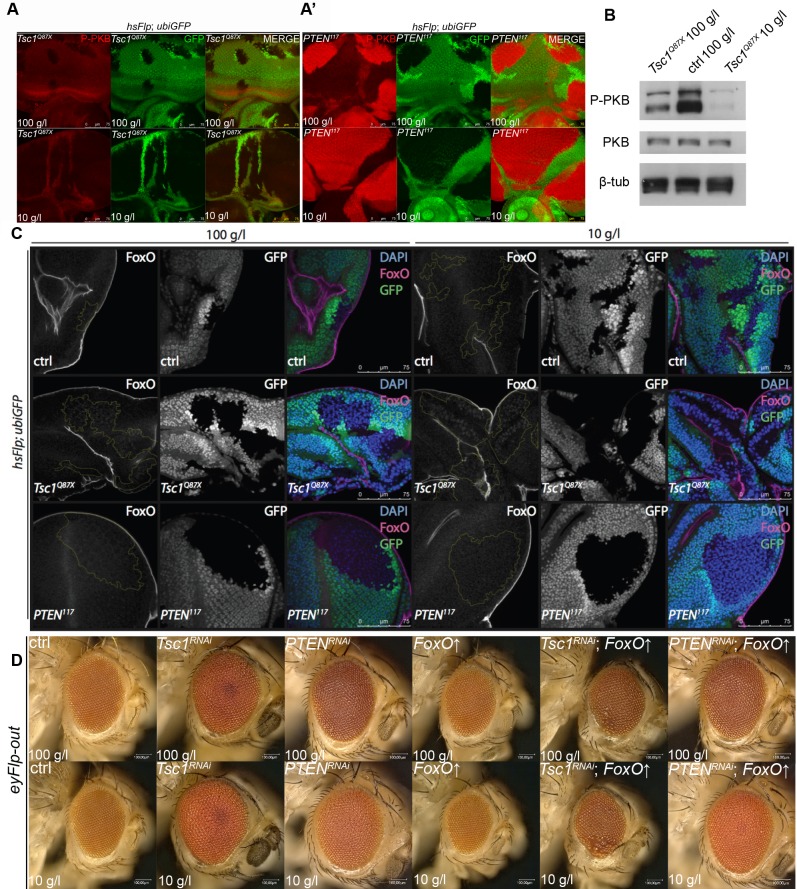
PKB-mediated inhibition of FoxO is lost in *Tsc1* mutant cells under NR. **(A)** Phospho-PKB staining (in red) of eyes discs with hsFlp *Tsc1* and *PTEN* **(A’)** mutant clones (marked by the absence of GFP) dissected from larvae reared on normal food and NR. Scale bars are 75 μm. **(B)** Western blot analysis of proteins extracted from the control or *Tsc1* mutant eye imaginal discs. The extremely small size of the control eye discs from animals reared on NR precluded the Western analysis. Total levels of PKB were unaltered. β**-**tubulin served as loading control. **(C)** FoxO staining (in magenta) of eyes discs with hsFlp control, *Tsc1* and *PTEN* clones (marked by the absence of GFP) dissected from larvae reared on normal food and NR. Scale bars are 75 μm. **(D)** Eyes bearing control, *Tsc1* knockdown or *PTEN* knockdown tissue, with or without the overexpression of FoxO, of animals reared on normal food or NR. Scale bars are 100 μm.

Overgrowth of epithelial cells mutant for *PTEN* depends on the activation of PKB under NR [18]. To investigate the role of PKB signaling in overgrowth of *Tsc1* mutant cells and to compare the activation status of PKB in *Tsc1* and *PTEN* mutant tissue, we performed phospho-PKB antibody immunostainings on discs bearing hsFlp *Tsc1* and *PTEN* mutant clones (Fig 4A and 4A'). Phospho-PKB levels were strongly elevated at the membrane in *PTEN* mutant clones. By contrast, the phospho-PKB signal was decreased in *Tsc1* mutant clones compared to the surrounding tissue under both food conditions. This is consistent with previous reports stating that PKB activity is downregulated by a negative feedback loop in the context of elevated TORC1 signaling under normal conditions [7, 17]. These results were confirmed by Western blot analyses for phospho-PKB and total PKB on *Tsc1* mutant discs. Phospho-PKB levels were consistently reduced under both conditions in the mutant discs as compared to control discs, with no observable change in total PKB levels. The decrease in PKB phosphorylation (and thus activity) was more pronounced in the *Tsc1* mutant tissue under NR (Fig 4B).

One of the key targets of PKB is the transcription factor FoxO. Activated PKB phosphorylates FoxO resulting in its cytoplasmic retention and inhibition of FoxO-dependent transcription of growth-inhibitory genes [27]. As shown previously [8], FoxO is localized to the nucleus in *Tsc1* mutant cells because of reduced PKB signaling. The nuclear intensity of FoxO was further increased upon NR (Fig 4C) only in *Tsc1* mutant cells, and could not be observed in control or *PTEN* mutant tissue. Overexpression of FoxO suppressed the overgrowth of *Tsc1* knockdown eyes, which was accompanied by partial loss of ommatidia. Thus, *Tsc1* mutant cells are highly susceptible to the growth-inhibitory activity of FoxO (Fig 4D, S6 Fig).

### FoxO restricts the proliferative potential of *Tsc1* mutant cells under NR

**Fig 5:**
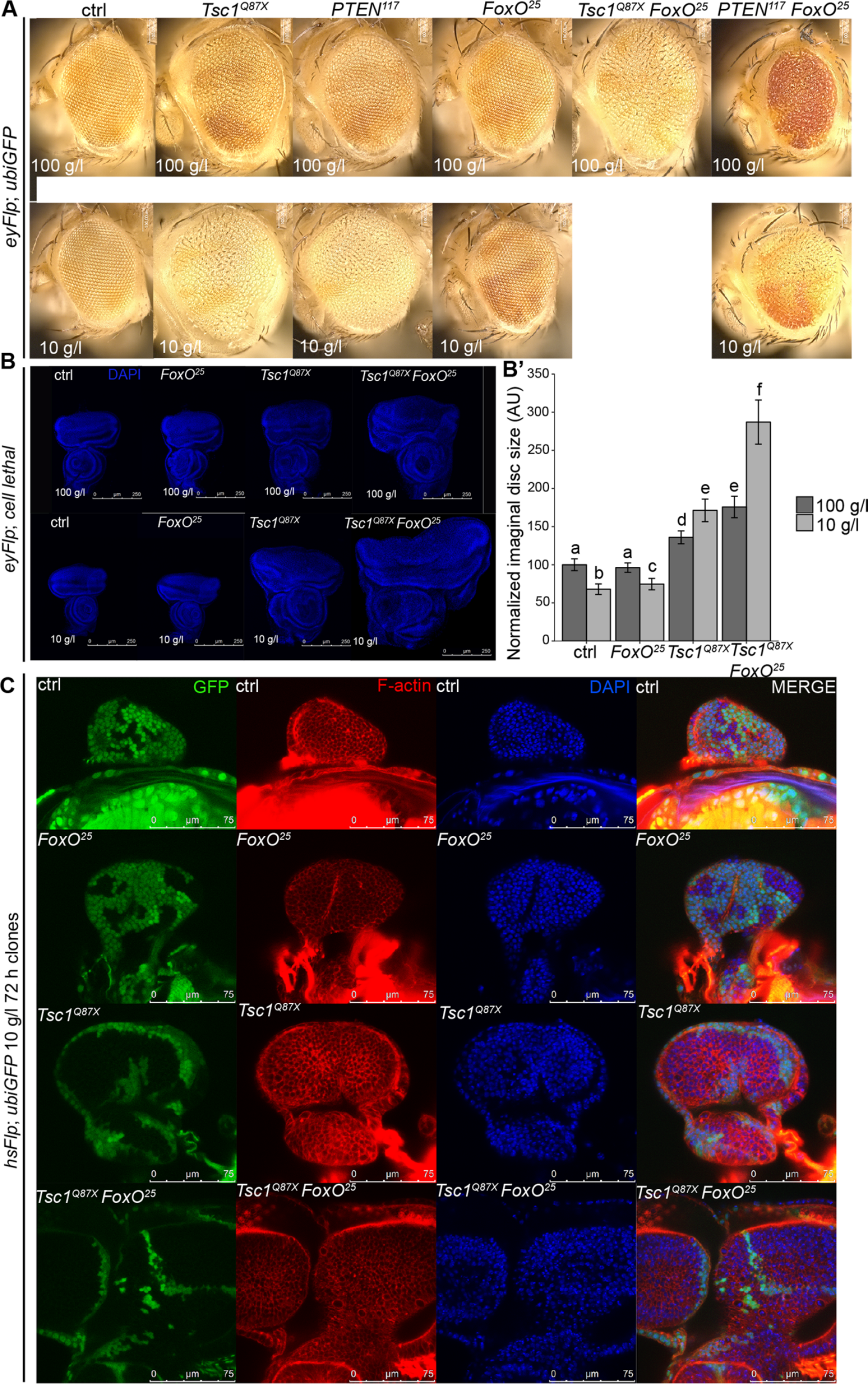
Loss of FoxO function leads to enhancement of *Tsc1* overgrowth under NR. **(A)** Eyes bearing eyFlp control, *Tsc1* or *PTEN* mutant clones (marked by the absence of pigmentation), with or without *FoxO* mutation of animals reared on normal food or NR. Removal of FoxO caused lethality of *Tsc1* mutant animals under NR. **(B)** Eye discs with control, *FoxO*, *Tsc1* or *Tsc1 FoxO* mutant tissue dissected from larvae reared on normal food or NR; **(B’)** quantification of eye disc size. Scale bars are 250 μm. **(C)** Nuclear (DAPI, blue) and cell membrane (phalloidin, red) staining in eye discs with hsFlp control, *FoxO*, *Tsc1* or *Tsc1 FoxO* clones (marked by the absence of GFP) dissected from larvae reared on NR. Scale bars are 75 μm.

Despite FoxO being dispensable for tissue growth under normal conditions, its role in the resistance to amino acid starvation [28] and in restricting tissue overgrowth upon high TORC1 signaling [8, 29] is well-established. Since *Tsc1* mutant cells also displayed elevated nuclear FoxO levels under NR, we were prompted to investigate the combined scenario of high TORC1 signaling, loss of FoxO function, and starvation. We compared the effects of loss of FoxO function in eyFlp control clones and in clones mutant for *Tsc1* or *PTEN* in animals reared on normal food and NR (Fig 5A). Consistent with previous reports [29], loss of FoxO did not impact the growth of control clones, and it partially reduced the overgrowth of *PTEN* mutant clones under both conditions. Intriguingly, removal of FoxO enhanced *Tsc1* mutant clone overgrowth on normal food and caused lethality of late 3rd instar larvae on NR. We then used the eyFlp cell lethal system to assess the growth of *Tsc1 FoxO* double mutant tissues earlier in development (Fig 5B and 5B’). On normal food, *Tsc1 FoxO* double mutant discs were consistently enlarged compared to *Tsc1* or *FoxO* mutant and control discs, and were of comparable size to *Tsc1* mutant discs under NR. Astonishingly, NR massively exacerbated the overgrowth of the double mutant discs that were almost 2.5 times larger than *Tsc1* mutant discs under the same conditions.

In mammals, FoxO has been shown to regulate proliferation via transcriptional induction of genes involved in cell cycle arrest [30]. The massive overgrowth of *Tsc1 FoxO* double mutant tissues upon NR could be caused by an increased proliferation rate or by reduced apoptosis (see below). We analyzed hsFlp clones in eye discs early in larval development on NR (Fig 5C). *FoxO* mutant clones grew roughly to the same extent as the adjacent wild-type twin clones, and the discs were of similar size as the ones with control clones. In contrast, *Tsc1* mutant clones were bigger than the wild-type twin spots and caused overgrowth of the discs. Loss of FoxO in *Tsc1* mutant cells dramatically increased the size of the discs and clones (due to more cells), causing concomitantly reduced growth of the surrounding tissue. The severe growth advantage on NR was confirmed by analyzing the adult eyes (S7A Fig). We also analyzed hsFlp clones induced later in development to quantify the effects on cell size and number (S7B and S7B’ Fig). The size of the double mutant clones was consistently larger than that of the single mutant ones (S7C’ Fig) but the cell density remained unchanged (S7C‴ Fig), indicating that the enhanced *Tsc1 FoxO* overgrowth is due to increased cell number.

FoxO activity is inhibited by PKB signaling [27]. However, apart from FoxO, PKB phosphorylates many other substrates that also contribute to cell survival, growth, and proliferation [27]. To investigate the role of PKB signaling in starvation-induced overgrowth of *Tsc1* mutant tissue, we generated eyFlp clones lacking *Tsc1*, *PKB*, *FoxO* and their double and triple mutant combinations, and analyzed their sizes in adult eyes (S8A and S8A’ Fig). Clonal loss of *PKB* on normal food reduced the eye size to the same extent as NR does in control eyes. The loss of *PKB* also suppressed the overgrowth of *Tsc1* mutant clones under both food conditions. Confirming the rationale that loss of *PKB* results in enhanced activity of FoxO that limits *Tsc1* overgrowth, the loss of *FoxO* in *Tsc1 PKB* double mutant clones not only restored overgrowth, but also enhanced it compared to the *Tsc1* mutant clones. Similar results were observed for clones in the eye discs (S8B Fig). Since the loss of *PKB* did not enhance the overgrowth of *Tsc1 FoxO* mutant clones, it is unlikely that other components downstream of PKB, apart from FoxO, play major roles in restricting the *Tsc1* loss-of-function overgrowth. Our observations rather suggest that other PKB targets promote growth. Moreover, FoxO itself does not modulate PKB signaling within *Tsc1* mutant tissue, as PKB signaling was downregulated to the same extent in *Tsc1* and *Tsc1 FoxO* mutant clones (S8C Fig). This excludes the possibility that a negative feedback regulation from FoxO on PKB contributes to the *Tsc1 FoxO* double mutant overgrowth, but rather indicates that FoxO target genes are limiting the overgrowth.

### *Tsc1 FoxO* double mutant cells are highly susceptible to cell death

**Fig 6:**
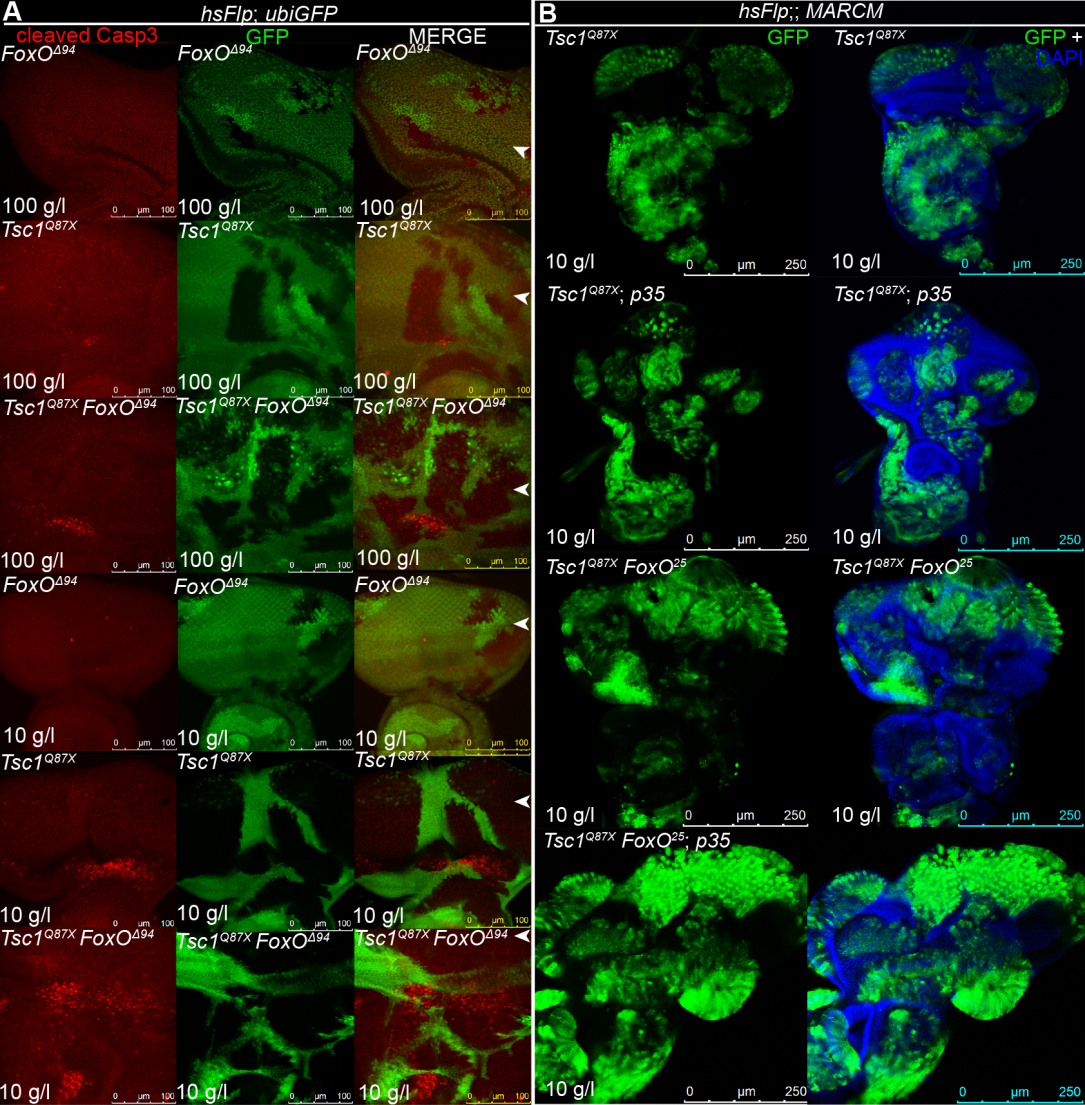
Loss of FoxO increases susceptibility of *Tsc1* mutant cells to cell death. **(A)** Cleaved Caspase-3 antibody staining (in red) of eye discs with hsFlp *FoxO*, *Tsc1* or *Tsc1 FoxO* mutant clones (marked by the absence of GFP) dissected from larvae reared on normal food and NR. The position of the morphogenetic furrow is indicated with a white arrowhead. Note that the *FoxO* null allele *FoxO*^*Δ94*^ was used in this experiment. Similar results were obtained with the *FoxO*^*25*^ allele. Scale bars are 100 μm. **(B)** Eye discs bearing MARCM *Tsc1* or *Tsc1 FoxO* clones (marked by GFP), with or without the expression of anti-apoptotic p35, dissected from larvae reared on NR. Scale bars are 250 μm.

FoxO has been shown to regulate apoptosis in mammals by inducing expression of pro-apoptotic genes [27]. We hypothesized that loss of FoxO inhibits apoptosis and therefore enhances the overgrowth of *Tsc1* mutant tissue on NR, which is prone to cell death (as shown in Fig 2). To test this, we monitored cell death in hsFlp *FoxO*, *Tsc1* and *Tsc1 FoxO* mutant clones by staining for cleaved Caspase-3 (Fig 6A). No apoptosis was observed in *FoxO* mutant clones under both food conditions. Little apoptosis was seen in *Tsc1* mutant clones on normal food (as shown in Fig 2), with high levels in proliferating cells on NR. Astonishingly, removal of FoxO from *Tsc1* mutant clones did not rescue the dying cells; instead, the double mutant clones displayed increased levels of apoptosis under both food conditions. Blocking cell death specifically in the double mutant clones by expression of p35 exacerbated the overgrowth of mutant tissue, especially on NR (Fig 6B). Thus, loss of FoxO renders *Tsc1* mutant cells highly susceptible to apoptosis, suggesting that increased proliferation rate is the major contributor to the enhanced overgrowth of *Tsc1 FoxO* double mutant tissue.

### *Tsc1 FoxO* double mutant tissue displays severe morphological defects upon NR

**Fig 7:**
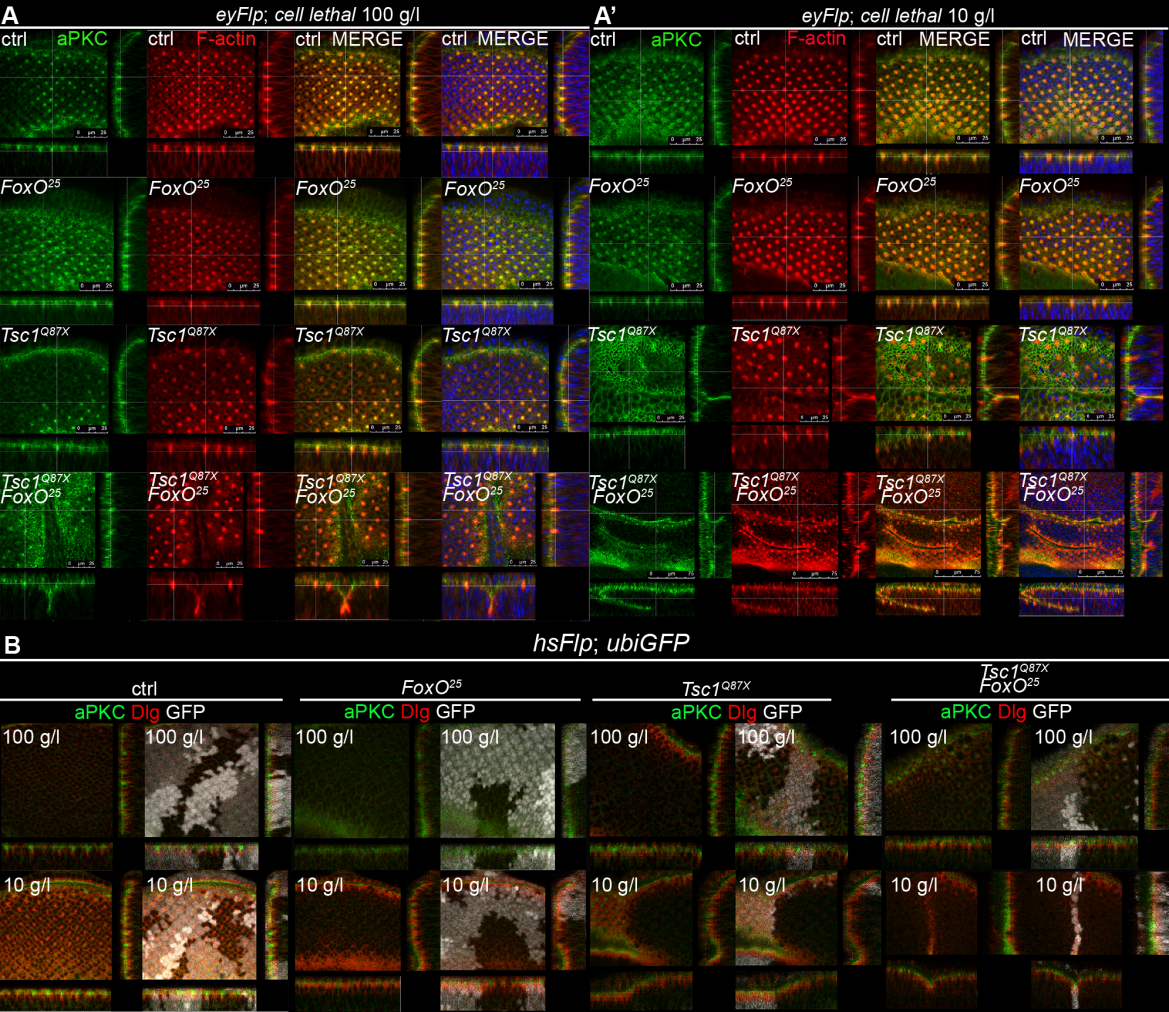
*Tsc1 FoxO* double mutant discs exhibit misfoldings due to massive overproliferation under NR. **(A)** aPKC (in green), phalloidin (in red) and DAPI (in blue) stainings in orthogonal sections of eye discs with control, *FoxO*, *Tsc1* and *Tsc1 FoxO* mutant tissue dissected from larvae reared on normal food and NR **(A’)**. Scale bars are 25 μm except for *Tsc1*^*Q87X*^ *FoxO*^*25*^ on NR, where they are 75 μm. A lower magnification was used to give a better overview of the misfolded tissue. **(B)** aPKC (in green) and Dlg (in red) stainings in orthogonal sections of eye discs with hsFlp control, *FoxO*, *Tsc1*, and *Tsc1 FoxO* mutant clones (marked by the absence of GFP) dissected from larvae reared on normal food and NR.

We observed severe morphological defects in the massively overgrown *Tsc1 FoxO* double mutant discs under NR. Since mutations in genes controlling apical-basal polarity cause a loss of epithelial organization and uncontrolled proliferation [31], we decided to investigate whether apical-basal polarity is disturbed in epithelial monolayers lacking Tsc1 and FoxO. We marked the apical membrane domain and F-actin by immunostaining control, *FoxO*, *Tsc1* and *Tsc1 FoxO* mutant discs with aPKC antibody and phalloidin, respectively (Fig 7A and 7A’). Control and *FoxO* mutant discs consisted of correctly organized epithelial monolayers under both food conditions. The structure of the *Tsc1* mutant tissue was also unaffected under normal food but displayed indentations in the epithelial monolayer under NR. Similar morphological defects were observed in *Tsc1 FoxO* double mutant tissue under normal food with severe distortions and multi-layering under NR. These defects were not due to the altered apical-basal polarity of the individual cells because aPKC and Dlg, markers of apical membrane and septate junction/basolateral membrane domains, respectively, were correctly localized within hsFlp clones of all genotypes embedded in eye epithelia under both food conditions (Fig 7B). We further analyzed this phenotype using the neuron-specific marker Elav together with phalloidin (S9 Fig). Elav protein distribution and actin organization were severely disturbed in *Tsc1 FoxO* double mutant discs under NR. We hypothesize that the morphological defects in *Tsc1* mutant tissue upon loss of FoxO are due to massive overproliferation that creates misfoldings of the epithelium.

### Loss of FoxO function results in precocious differentiation in tissues with high TORC1 activity on NR

**Fig 8:**
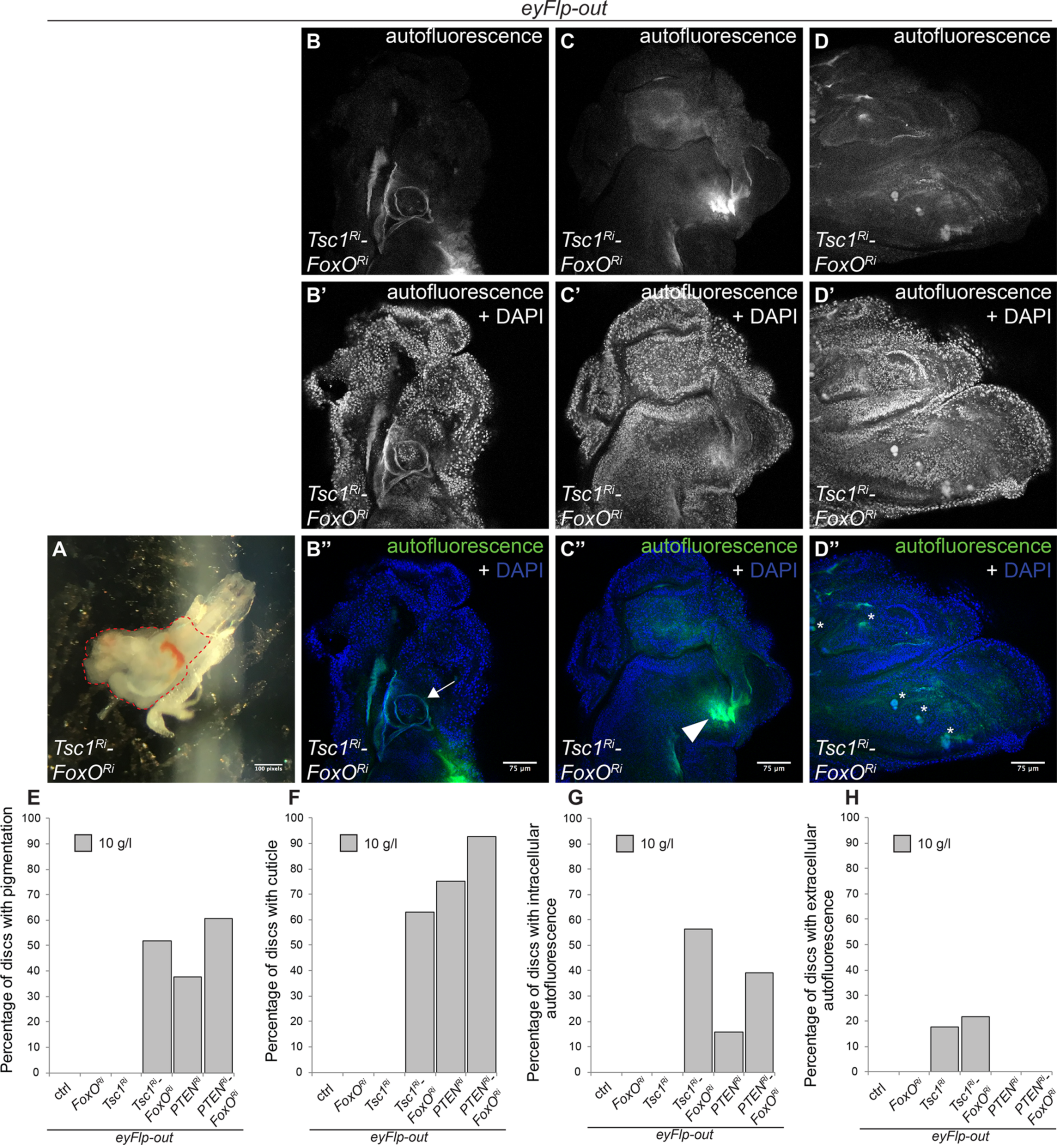
Concomitant loss of *FoxO* and high TORC1 activity causes precocious differentiation under NR. **(A)** Pigmentation in eye discs with *Tsc1* and *FoxO* co-knockdown (disc is marked by dashed-red line, still attached to mouth hook) dissected from larvae reared under NR. Scale bar is 0.5 mm. **(B-D)** Autofluorescence seen in the green and blue **(B’-D’)** channels for **(B”)** cuticle (marked by arrow), **(C”)** intracellular autofluorescence (marked by arrowhead) and **(D”)** extracellular autofluorescence (marked by asterisks). Scale bars are 75 μm. Quantification of percentage of discs with **(E)** pigmentation, **(F)** cuticle, **(G)** intracellular autofluorescence, and **(H)** extracellular autofluorescence. n = 6–10 for control and *FoxO* knockdown, and n > 30 for the remaining cases.

Upon NR, the larval growth period varies greatly. L3 larvae can be found from 148 to 408 hours after egg laying. To analyze the effect of this extended growth phase, we generated various knockdowns using the eyFlp-out system and dissected eye discs every 24 hours. Remarkably, *Tsc1* and *Tsc1 FoxO* knockdown discs reached up to eight times the size of control and *FoxO* knockdown discs (S10A Fig). These discs did not conform to the flat epithelial architecture of the control discs and displayed outgrowths protruding from the surface on later days of dissection (S10B Fig). *PTEN* knockdown discs grew three times the size of control discs and maintained this size for the remaining larval period. Similar results were observed in eye discs composed of cells mutant for the respective genes. Unexpectedly, signs of precocious differentiation were observed in the *PTEN*, *PTEN FoxO* and *Tsc1 FoxO* knockdown discs (Fig 8). These discs showed pigmentation (Fig 8A and 8E) and cuticle formation (Fig 8B–8H) starting from 264 hours after egg laying. Three kinds of autofluorescent patterns were noticed that were categorized as cuticular, intracellular, and extracellular (S1–S4 Videos). We hypothesize that the intracellular autofluorescence is due to the unsecreted autofluorescent cuticle, and that the extracellular autofluorescence stems from the proteins yet to form the cuticle. The extracellular autofluorescence was specific to discs with *Tsc1* and *Tsc1 FoxO* knockdowns. No signs of differentiation were found in control or *FoxO* knockdown discs. Differentiation was also specific to NR, as no pigmentation was observed in discs dissected from larvae on normal food, even with prolonged development at 18°C. Thus, FoxO not only plays an important role in restricting growth of cells with high TORC1 activity but it also prevents precocious differentiation.

Interestingly, eye discs with a *Tsc1 FoxO* co-knockdown reached a rather constant size irrespective of the highly variable larval sizes on NR (S10C Fig), pointing to an organ-autonomous size control mechanism.

## Discussion

PTEN and Tsc1/2 are major tumor suppressors in the PI3K-PKB-TORC1 signaling network. Whereas *PTEN* mutations can lead to malignant cancers, mutations in *Tsc1/2* mostly cause benign hamartomas. In this study, we mimicked pre-cancerous situations in the larval imaginal discs of *Drosophila* to investigate the mechanisms responsible for the pronounced phenotypic differences. By comparing the growth behavior of clonal populations lacking either PTEN or Tsc1 under starvation conditions, we identified a key role for the transcription factor FoxO in restricting proliferation of *Tsc1* mutant cells.

Tumors with high PI3K activity have been shown to be starvation resistant. Our results demonstrate that similar to the activation status of PI3K, high TORC1 activity due to the loss of Tsc1 also confers a growth advantage to cells under NR. Populations of *Tsc1* mutant cells embedded in heterozygous epithelia are capable of ousting the neighboring cells when nutrients are limiting. The *Tsc1* mutant cells overproliferate within the margins of the organ but, due to increased apoptosis limiting cell number, contribute to the overall increase in organ size in a hypertrophic manner. The ousting of the neighboring cells is not due to classical cell competition, as the cells surrounding the clones do not show signs of apoptosis. Their growth is rather inhibited, probably because of competition for nutrients. This illustrates how starvation exerts selective pressure on different cellular populations, and creates conditions permissive for tumor expansion. *Tsc1* mutant cells are insensitive to NR at the level of TORC1 activation, as the phosphorylation of S6K remains unchanged. This may also explain why the size of *Tsc1* mutant cells–in contrast to heterozygous and wild-type cells–is not reduced under NR. On the contrary, *Tsc1* mutant cells increase their hypertrophic potential and grow even larger upon NR. This hypertrophic phenotype is in sharp contrast to the previously described shift from hypertrophic to mainly hyperplastic growth induced by the loss of PTEN in mitotic cells under NR [18]. We note that the activation status of TORC1, which is reduced in *PTEN* mutant cells upon NR but remains constantly high in *Tsc1* mutant cells, parallels the observed cell size.

Our genetic analyses point to the differential activation status of PKB and its target FoxO as the primary cause for the differential growth behavior of *PTEN* and *Tsc1* mutant cells. FoxO activity is largely inhibited in *PTEN* mutant tissue upon NR due to elevated PKB signaling. Consistently, loss of FoxO function does not considerably impact *PTEN* mutant tissue overgrowth in *Drosophila* epithelia. By contrast, *Tsc1* mutant cells display low PKB activity and are highly sensitive to elevated levels of FoxO. The mutant clones collapse and the overgrowth is completely suppressed upon overexpression of FoxO. This is in line with previous reports that FoxO-mediated transcription restricts the overgrowth caused by the loss of Tsc1 in *Drosophila* eye epithelia under normal conditions [8]. Our study reveals that FoxO function is rate-limiting for restricting the proliferation of *Tsc1* mutant cells upon NR. Loss of FoxO does not enhance the *Tsc1* mutant overgrowth by reducing apoptosis of cells–*Tsc1 FoxO* mutant cells are highly susceptible to cell death–but by increasing proliferation rates. Removal of FoxO from *Tsc1* mutant tissue under NR results in the induction of massive hyperplasia and malformations, disrupting the proper architecture of the epithelium.

Upon prolonged starvation, loss of Tsc1 in the developing eyes resulted in eye discs six times as large as the control discs. Concomitant loss of FoxO generated discs that sometimes occupied two-thirds of the larva and severely compromised the surrounding larval tissues. We did not observe a metastatic behavior of the *Tsc1* mutant cells. By contrast, tissues with loss of FoxO function and high TORC1 activity (*PTEN*, *PTEN FoxO* and *Tsc1 FoxO*) revealed signs of precocious differentiation, such as pigmentation and formation of adult cuticle. The formation of adult structures within mitotically active epithelia is reminiscent of teratoma formation (although we did not observe the formation of structures from unrelated organs). A pulse of the steroid hormone ecdysone initiates metamorphosis after the critical weight is reached [32]. FoxO has been shown to inhibit ecdysone biosynthesis by directly interacting with a part of the ecdysone receptor, Ultraspiracle [33]. The observation of precocious differentiation suggests that cells with high TORC1 activity that have lost the function of FoxO have a lower threshold for ecdysone response, possibly due to a similar inhibitory action of FoxO on the ecdysone receptor complex. As a consequence, differentiation occurs sporadically in these cells. Whether there are higher levels of ecdysone in the hemolymph due to non-cell autonomous effects on the prothoracic gland remains to be examined.

The similar behaviors of *PTEN* and *Tsc1 FoxO* mutant tissues suggest that the loss of FoxO contributes to the transformation of benign growth of *Tsc1* mutant hamartomas into malignant tumors. The nutrient fluctuations in the microenvironment of hamartomas may also enhance tumor progression, as is seen by the dramatic overgrowth of the *Tsc1 FoxO* mutant tissue upon NR. Indeed, although malignant tumors are rare in tuberous sclerosis syndrome, conversion of renal cell carcinomas to malignancies were observed, and FoxO was shown to be expressed in benign but not in aggressive renal tumor [34]. Thus, the differential strength of PKB signaling opposing the activity of FoxO in the context of loss of Tsc1 and PTEN points to the FoxO activation status as the key determinant of the malignant potential of tumors caused by the loss of these tumor suppressors. It will be interesting to identify the FoxO target genes responsible for restricting the proliferative potential of *Tsc1* mutant cells.

## Materials and methods

### Fly media and stock keeping

1 liter *Drosophila* medium contains 100g of fresh yeast, 55 g cornmeal, 10 g wheat flour, 75 g sugar and 8 g bacto agar, here referred to as “normal food” or “100 g/l yeast” food. Starvation food was prepared by reducing the amount of yeast to the indicated values (50g/l, 30g/l, 20g/l, 10g/l, or 5g/l) without altering the other components. All crosses and experiments were performed at 25°C in non-crowding conditions.

### Mutants and transgenes

For clonal analyses and genetic interaction studies, *FRT82 Tsc1*^*Q87X*^ [16], *FRT82 Rheb*^*2G5*^ [35], *FRT82 PKB*^*1*^ [36], *FRT82 FoxO*^*25*^ [29], *FRT82 FoxO*^*Δ94*^ [37], *PTEN*^*117*^ *FRT40* [38], *UAS*-*4E*-*BP*^*WT*^, *UAS*-*4E*-*BP*^*AA*^ [26, 40], *UAS-cherry-dFoxO* (N. Alic), or combinations thereof were used. An isogenic *FRT82* line served as control in all experiments. *UAS-p35* [39] was used to block cell death. TORC1 signaling was activated by means of *EP-Rheb* [35]. Knockdowns were generated using the lines *UAS*-*Tsc1*^*Ri*^ (BDSC 31039), *UAS-Tsc2*^*Ri*^ (BDSC 34737), *UAS*-*PTEN*^*Ri*^ (VDRC 101475), *UAS*-*S6K*^*Ri*^ (VDRC 18126), *UAS*-*Raptor*^*Ri*^ (VDRC), and *UAS*-*FoxO*^*Ri*^ (VDRC 107786). *UAS-lacZ* served as control, except for the differentiation experiment where *UAS-CG33920*^*Ri*^ (VDRC 103447) was used as a control. A full list of genotypes is provided in the supplementary material (S1 Genotypes).

### Food experiments

Flies were crossed for two days in standard rearing vials, transferred to egg laying chambers, and allowed to lay eggs on agar plates containing yeast paste for 11 hours. For induction of eyFlp/FRT clones, eggs were immediately distributed to different food conditions, whereas for induction of hsFlp/FRT clones, first instar larvae were allowed to hatch and heat-shocked before distribution.

### Clonal studies

Temporally controlled clones, negatively marked by the absence of GFP or positively marked by the presence of GFP, were generated using the hsFlp/FRT and hsFlp/MARCM systems, respectively. Clones were induced in first instar larvae by a heat shock (15 min, 37°C) 38h after egg laying (AEL), and eye discs were dissected from third instar larvae before the wandering stage or at the indicated time point. Pupal retinae were dissected 46h after puparium formation. For induction of 72h and 48h hsFlp clones, eggs were collected in a short time window (4 hours) and heat shock (13min, 37°C) was induced 31h and 55h AEL, respectively. For adult eye analyses, clones negatively marked by the absence of pigmentation were generated by using the eyFlp/FRT system. For eye discs entirely composed of the mutant or knockdown tissue, clones were induced with the eyFlp/FRT cell lethal system or by using the eyFlp-out system, respectively. Larvae were dissected at indicated time points, or left to develop to adults for analysis of the eyes. To get tangential eye sections, adult heads were halved, fixed using OsO_4_, dehydrated with acetone, and embedded in Spurr. 2 μm sections were prepared using a microtome (Reichert-Jung 2050).

### Phenotypic analyses

All the phenotypic analyses on adult flies were performed on females. For the light micrographs of the adult eyes, a KEYENCE VHX1000 digital microscope was used. For ommatidia analyses, a Jeol JSM-6360LV scanning electron microscope (SEM) was used. The sizes of the eyes and ommatidia were defined by measuring the pixels of their area in Photoshop CS6. For DIC and confocal images of eye discs and larvae, a Leica SP2 confocal laser-scanning microscope was used. Eye sections were imaged on Zeiss Axiophot. 3D reconstruction of stacks was done using IMARIS.

### Statistical analyses

In all quantifications (S1 Datasets), Student’s t-test (two tailed) was used to test for significance. In each experiment, at least 10 individuals were measured for each genotype (unless otherwise stated). Significance (p < 0.05, pairwise t-tests) is indicated in the figures using letters such that conditions denoted by the same letter are statistically not different from each other. Error bars represent standard deviations.

### Immunochemistry and Western blotting

Larval imaginal discs were fixed in 4% paraformaldehyde (PFA, 30min, room temperature), permeabilized in 0.3% Tween in PBS (PBT, 15min, room temperature), blocked in 2% NDS in 0.3% PBT (1h, room temperature), incubated with primary antibodies overnight (4°C), washed three times in 0.3% PBT, and incubated with the secondary antibodies (1h, room temperature). The nuclei were visualized with DAPI in 0.3% PBT (1:2000, 15min, room temperature). F-actin was visualized with phalloidin. Antibodies used were: rabbit α*-Drosophila* phospho-PKB Ser505 (1:300, Cell Signaling), rabbit α-cleaved caspase 3 (1:300, Cell Signaling), rabbit α-aPKC (1:500, Promega), mouse α-Dlg (1:50, DSHB), α-Elav (DSHB), rabbit α-dFoxO (1:500, gift from Marc Tatar), Cy3-coupled α-mouse or α-rabbit IgG (1:300, Amersham), and Alexa Fluor 633 (1:500, Thermo Fisher Scientific). All the dilutions of antibodies were made in 2% NDS in 0.3% PBT blocking solution.

Western blots on third instar eye imaginal discs were performed according to standard protocols. Antibodies were α-*Drosophila* phospho-PKB Ser505 (1:1000, Cell Signaling), α*-*PKB (1:1000, Cell Signaling), α-phospho-S6K (1:1000, Cell Signaling), α-S6K (1:2000, our own antibody), α-β**-**tubulin (1:10000, Sigma), HRP-conjugated α-mouse and α-rabbit IgG (1:10000, Amersham).

## Supporting information

S1 Fig *Tsc2* mutant cells have a growth advantage under NR. **(A)** Eyes with control or *Tsc2* knockdown tissue of animals reared on normal food and NR (scale bars are 100 μm), **(A’)** corresponding eye discs (scale bars are 250 μm), and **(A”)** quantification of eye disc size.
(TIF)

S2 Fig Ommatidial architecture remains unchanged upon loss of *Tsc1* function. **(A)** Sections of eyes with hsFlp *Tsc1* mutant clones (marked by the absence of pigmentation) of animals reared on normal food and NR. **(B)** Phalloidin staining (in red) of pupal retinae with hsFlp *Tsc1* mutant clones (marked by the absence of GFP) of animals reared on normal food and NR. Scale bars are 25 μm.
(TIF)

S3 Fig Apoptosis in clones of *Tsc1* mutant cells counteracts overgrowth. **(A)** Eye discs bearing MARCM *Tsc1* mutant clones (marked by GFP), with or without the expression of anti-apoptotic p35, dissected from larvae reared on normal food or NR. Scale bars are 250 μm. **(B)** Cleaved Caspase-3 antibody staining (in red) of eye discs with hsFlp *Tsc1* mutant clones (marked by the absence of GFP) dissected from larvae reared on normal food or NR. Scale bars are 100 μm.
(TIF)

S4 Fig TORC1-dependent overgrowth of *Tsc1* mutant cells is not mediated by inactivation of 4E-BP. **(A)** Eye discs bearing MARCM control, *4E-BP*^*WT*^ or *4E-BP*^*AA*^ clones (marked by GFP), with or without *Tsc1* mutation, dissected from larvae reared on normal food and NR. Scale bars are 250 μm.
(TIF)

S5 Fig Activation of TORC1 by Rheb promotes tissue overgrowth on NR. **(A)** Eye discs with MARCM control or *EP-Rheb* clones (marked by GFP) dissected from larvae reared on normal food and NR. Scale bars are 250 μm. **(B)** Scanning electron micrographs of control or *EP-Rheb* eyes of animals reared on normal food and NR; **(B’)** quantification of eye size. Scale bars are 100 μm. **(C)** Cleaved Caspase-3 antibody staining (in red) of eye discs with MARCM *EP-Rheb* clones (marked by GFP) dissected from larvae reared on normal food or NR. Scale bars are 100 μm.
(TIF)

S6 Fig Overexpression of FoxO suppresses overgrowth caused by *Tsc1* knockdown. Quantification of eyes shown in Fig 4D.
(TIF)

S7 Fig Loss of FoxO enhances the proliferation of *Tsc1* mutant cells under NR. **(A)** Eyes with hsFlp control, *Tsc1*, *FoxO* and *Tsc1 FoxO* mutant clones (marked by the absence of pigmentation) of animals reared on normal food and NR. **(B, B’)** Eye discs with hsFlp *Tsc1* and *Tsc1 FoxO* clones (marked by the absence of GFP) dissected from larvae reared on NR 48 h after clone induction (scale bars are 75 μm), and the quantification of **(C)** eye disc area, **(C’)** mutant clone area, **(C”)** mutant clone area relative to the whole disc, and **(C‴)** mutant cell density (number of nuclei per arbitrary square).
(TIF)

S8 Fig Extent of the *Tsc1* mutant overgrowth is dependent on PKB-mediated inhibition of FoxO. **(A)** Eyes bearing eyFlp control, *Tsc1*, *PKB* or *Tsc1 PKB* mutant clones (marked by the absence of pigmentation), with or without *FoxO* mutation, of animals reared on normal food and NR; **(A’)** quantification of eye size. **(B)** Eye discs bearing eyFlp control, *PKB*, *FoxO* or *PKB FoxO* mutant clones (marked by the absence of GFP), with or without *Tsc1* mutation, dissected from larvae reared on normal food or NR. Scale bars are 250 μm. **(C)** Phospho-PKB staining (in red) of eyes discs with hsFlp *Tsc1*, *FoxO* or *Tsc1 FoxO* mutant clones (marked by the absence of GFP) dissected from larvae reared on normal food and NR. Scale bars are 75 μm.
(TIF)

S9 Fig *Tsc1 FoxO* double mutant discs display severe morphological defects and multi-layering under NR. Elav (in green), phalloidin (in red) and DAPI (in blue) stainings in orthogonal sections of eye discs with control, *FoxO*, *Tsc1* and *Tsc1 FoxO* mutant tissue dissected from larvae reared on normal food and NR. Scale bars are 50 μm.
(TIF)

S10 Fig Overgrowth of *Tsc1 FoxO* double mutant discs upon NR. **(A)** Comparison of development of eye discs with control, *FoxO*, *Tsc1* or *Tsc1 FoxO* mutant tissue, dissected at indicated time points from larvae reared on normal food and NR. White arrows indicate morphological defects. **(B)** DIC images of eye discs with *Tsc1 FoxO* mutant tissue dissected 11 days after egg lay from larvae reared on NR. White arrows indicate morphological defects. **(C)** Comparison of size of the eye discs with *Tsc1 FoxO* knockdown tissue (marked by GFP) to larval size for various larvae (n = 46) reared on NR and imaged 19 days AEL.
(TIF)

S1 Video Overgrowth of *Tsc1* knockdown disc on NR. Comparison of 3D reconstructions of control disc with *Tsc1*^*Ri*^ disc on NR.
(MP4)

S2 Video Overgrowth of *Tsc1 FoxO* co-knockdown disc on NR. Comparison of 3D reconstructions of control disc with *Tsc1*^*Ri*^ and *FoxO*^*Ri*^ disc on NR.
(MP4)

S3 Video Overgrowth of *PTEN* knockdown disc on NR. Comparison of 3D reconstructions of control disc with *PTEN*^*Ri*^ disc on NR.
(MP4)

S4 Video Overgrowth of *PTEN FoxO* co-knockdown disc on NR. Comparison of 3D reconstructions of control disc with *PTEN*^*Ri*^ and *FoxO*^*Ri*^ disc on NR.
(MP4)

S1 Datasets Tables containing all measurements and statistical analyses for Figs 1, 2, 3, 5, S1, S5, S6, S7 and S8. (XLSX)

S1 Genotypes Genotypes of flies used in this study. (PDF)
