# Supplementary figures and images for: FoxO restricts growth and differentiation of cells with elevated TORC1 activity under nutrient restriction
Source: PLoS Genet. 2018 Apr 20;14(4):e1007347. doi: 10.1371/journal.pgen.1007347 (PMC5931687; doi:10.1371/journal.pgen.1007347)

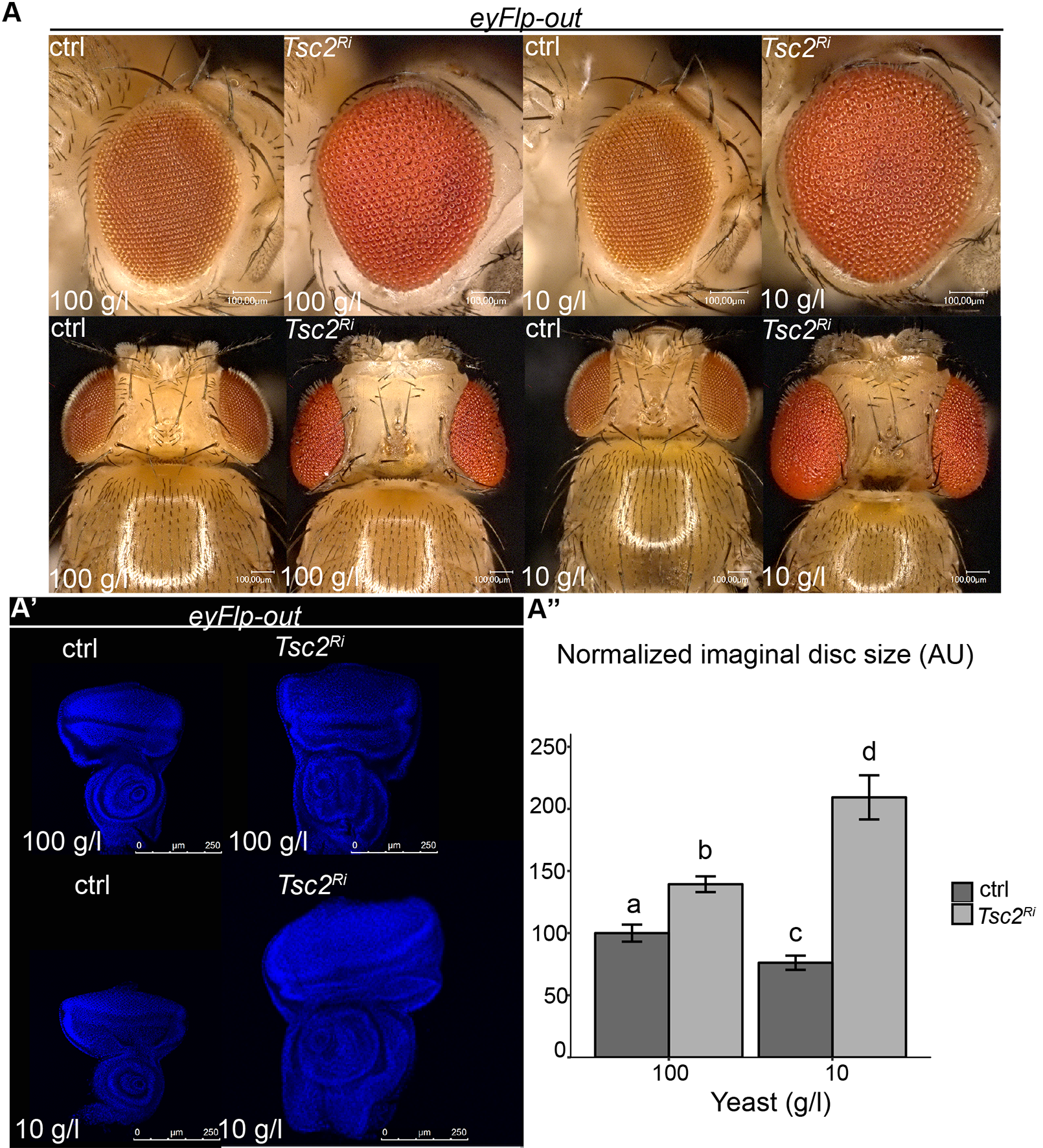

Supplement: S1 Fig — (A) Eyes with control or Tsc2 knockdown tissue of animals reared on normal food and NR (scale bars are 100 μm), (A’) corresponding eye discs (scale bars are 250 μm), and (A”) quantification of eye disc size. (TIF) [file pgen.1007347.s001.tif]

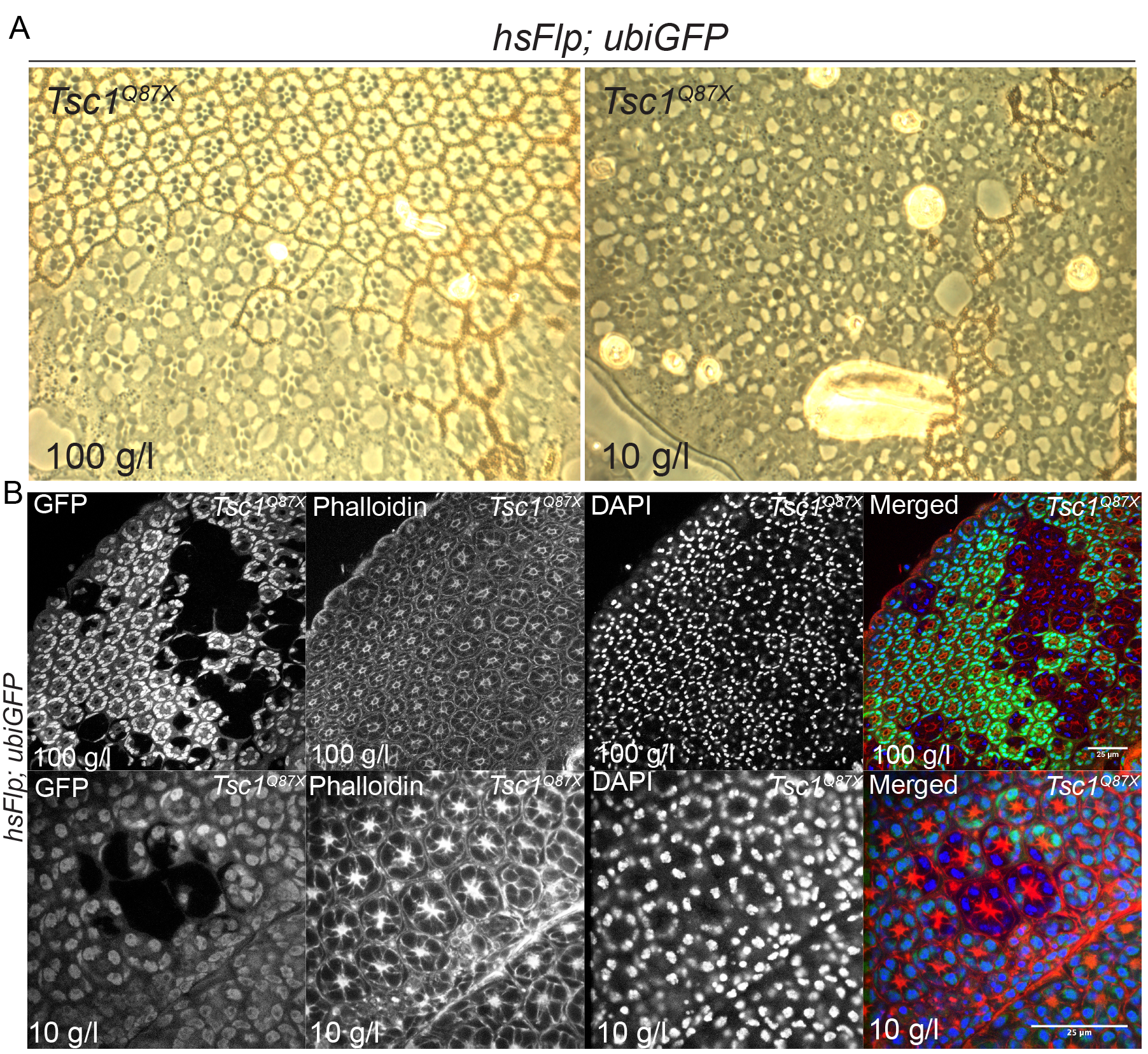

Supplement: S2 Fig — (A) Sections of eyes with hsFlp Tsc1 mutant clones (marked by the absence of pigmentation) of animals reared on normal food and NR. (B) Phalloidin staining (in red) of pupal retinae with hsFlp Tsc1 mutant clones (marked by the absence of GFP) of animals reared on normal food and NR. Scale bars are 25 μm. (TIF) [file pgen.1007347.s002.tif]

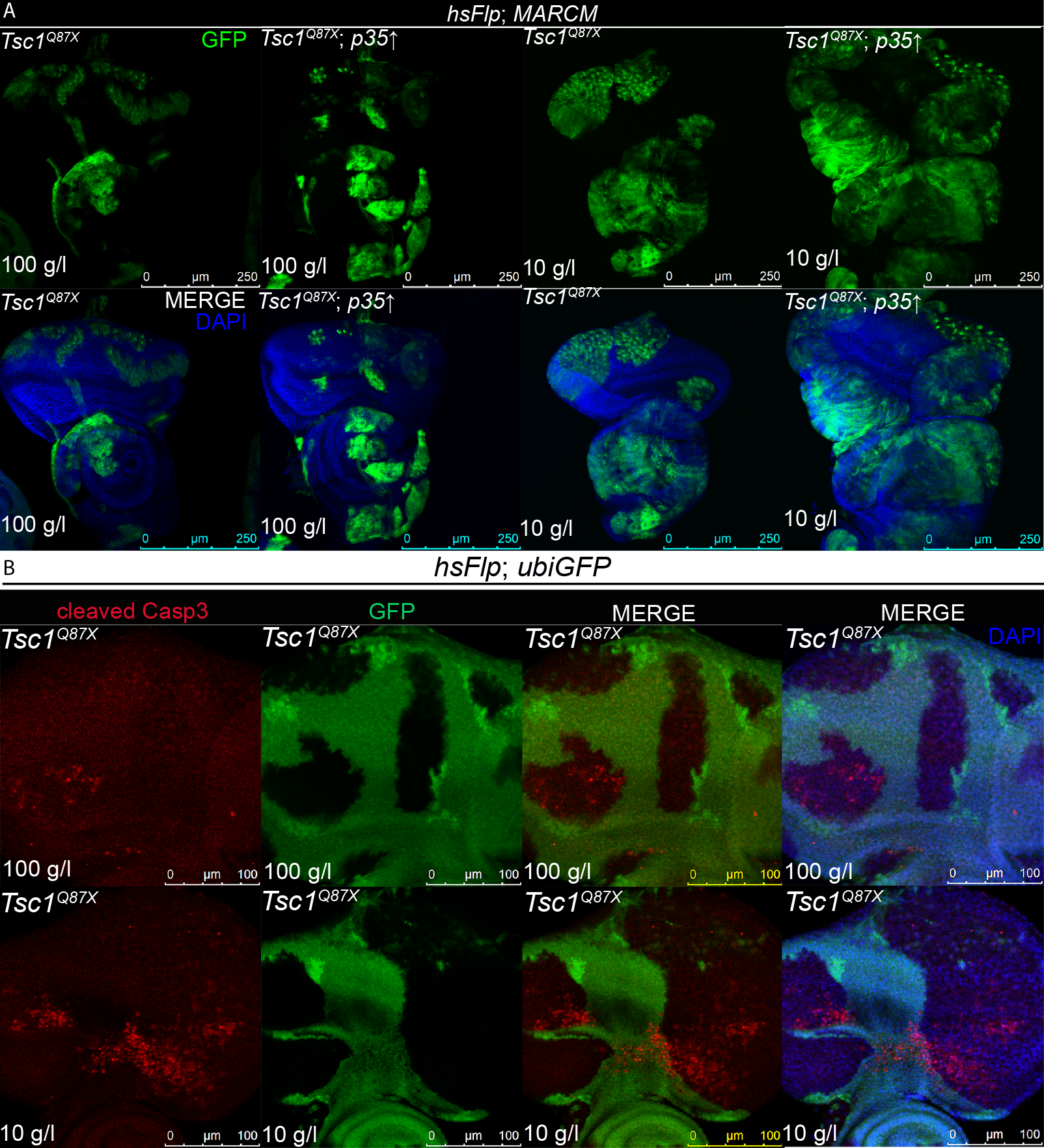

Supplement: S3 Fig — (A) Eye discs bearing MARCM Tsc1 mutant clones (marked by GFP), with or without the expression of anti-apoptotic p35, dissected from larvae reared on normal food or NR. Scale bars are 250 μm. (B) Cleaved Caspase-3 antibody staining (in red) of eye discs with hsFlp Tsc1 mutant clones (marked by the absence of GFP) dissected from larvae reared on normal food or NR. Scale bars are 100 μm. (TIF) [file pgen.1007347.s003.tif]

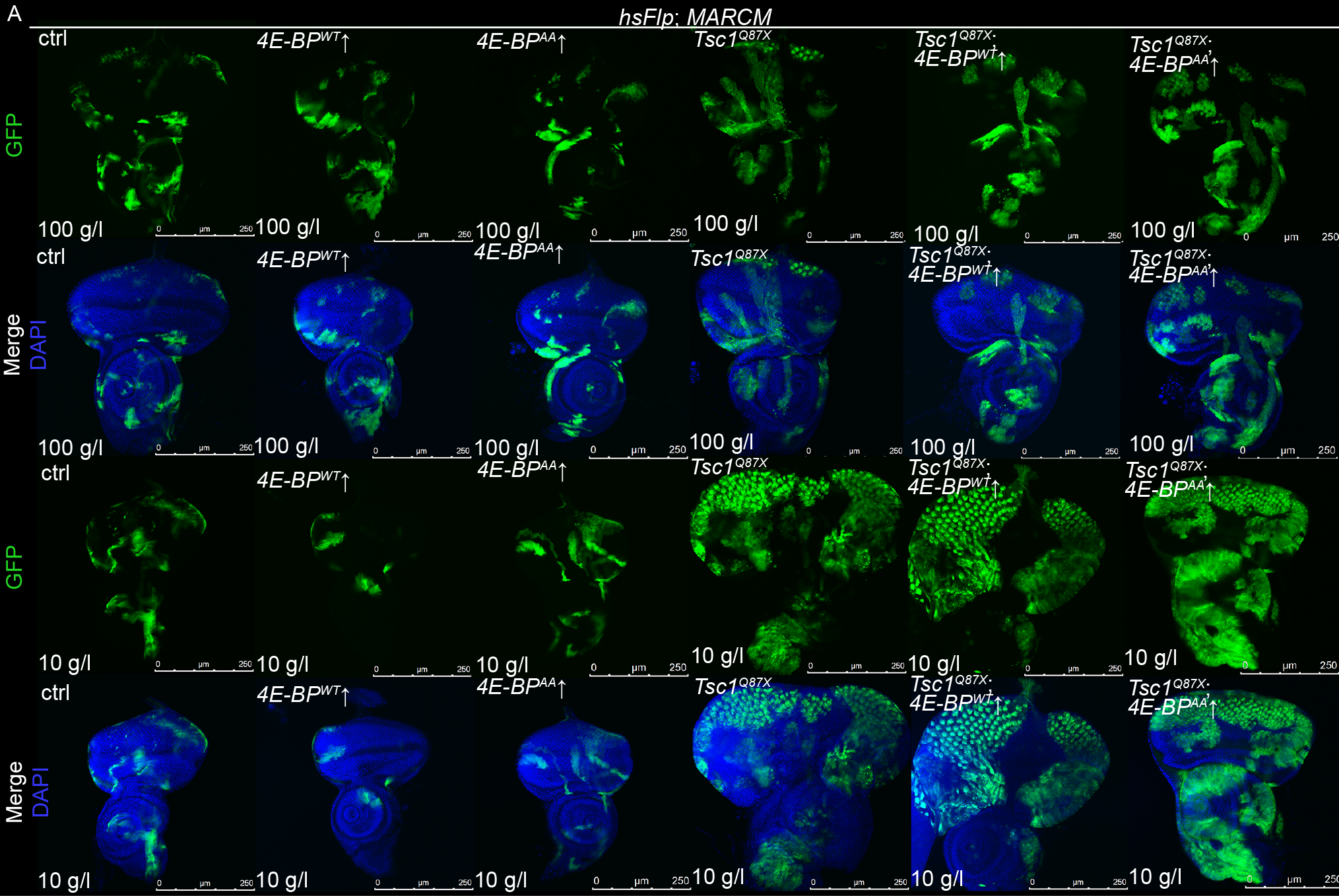

Supplement: S4 Fig — (A) Eye discs bearing MARCM control, 4E-BPWT or 4E-BPAA clones (marked by GFP), with or without Tsc1 mutation, dissected from larvae reared on normal food and NR. Scale bars are 250 μm. (TIF) [file pgen.1007347.s004.tif]

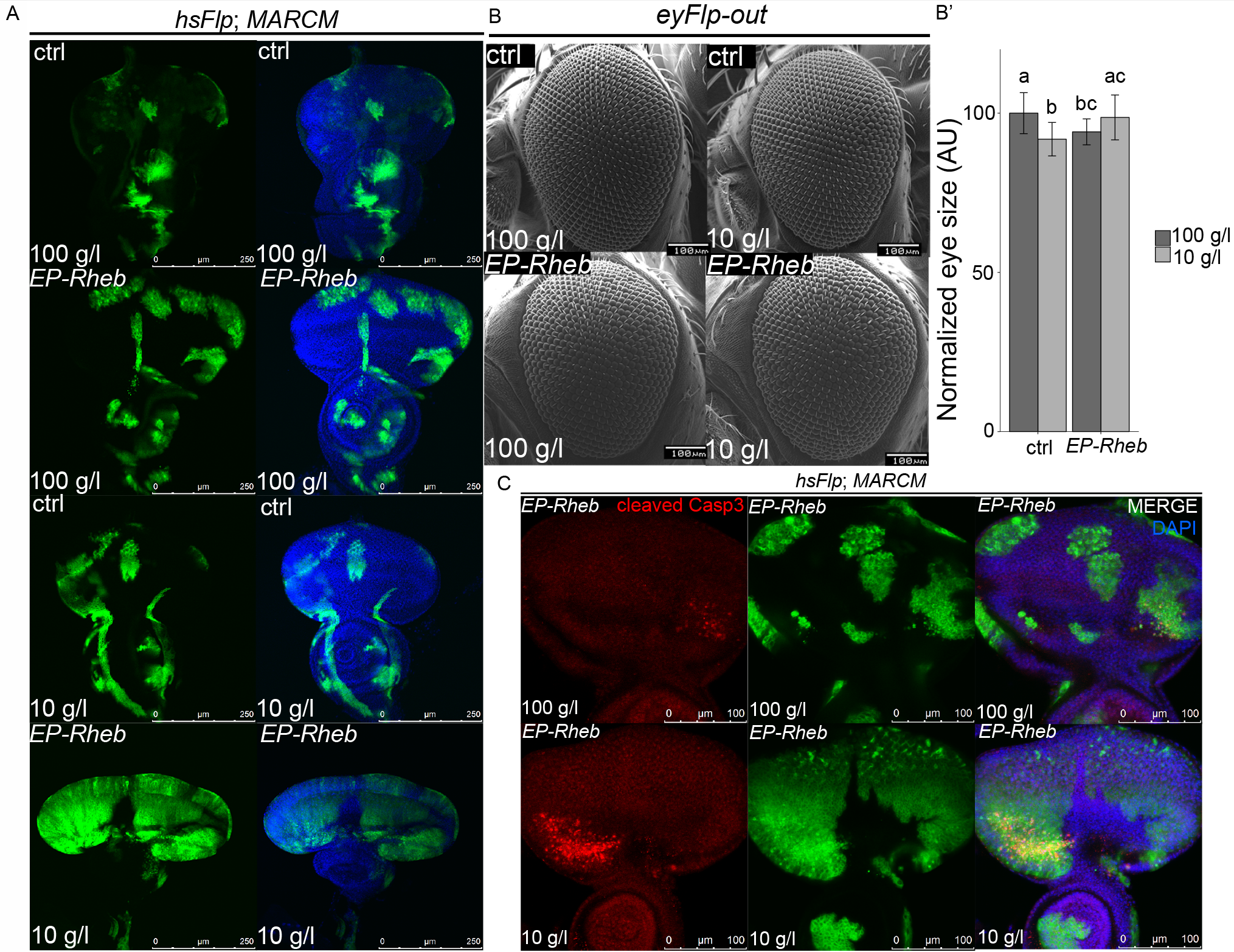

Supplement: S5 Fig — (A) Eye discs with MARCM control or EP-Rheb clones (marked by GFP) dissected from larvae reared on normal food and NR. Scale bars are 250 μm. (B) Scanning electron micrographs of control or EP-Rheb eyes of animals reared on normal food and NR; (B’) quantification of eye size. Scale bars are 100 μm. (C) Cleaved Caspase-3 antibody staining (in red) of eye discs with MARCM EP-Rheb clones (marked by GFP) dissected from larvae reared on normal food or NR. Scale bars are 100 μm. (TIF) [file pgen.1007347.s005.tif]

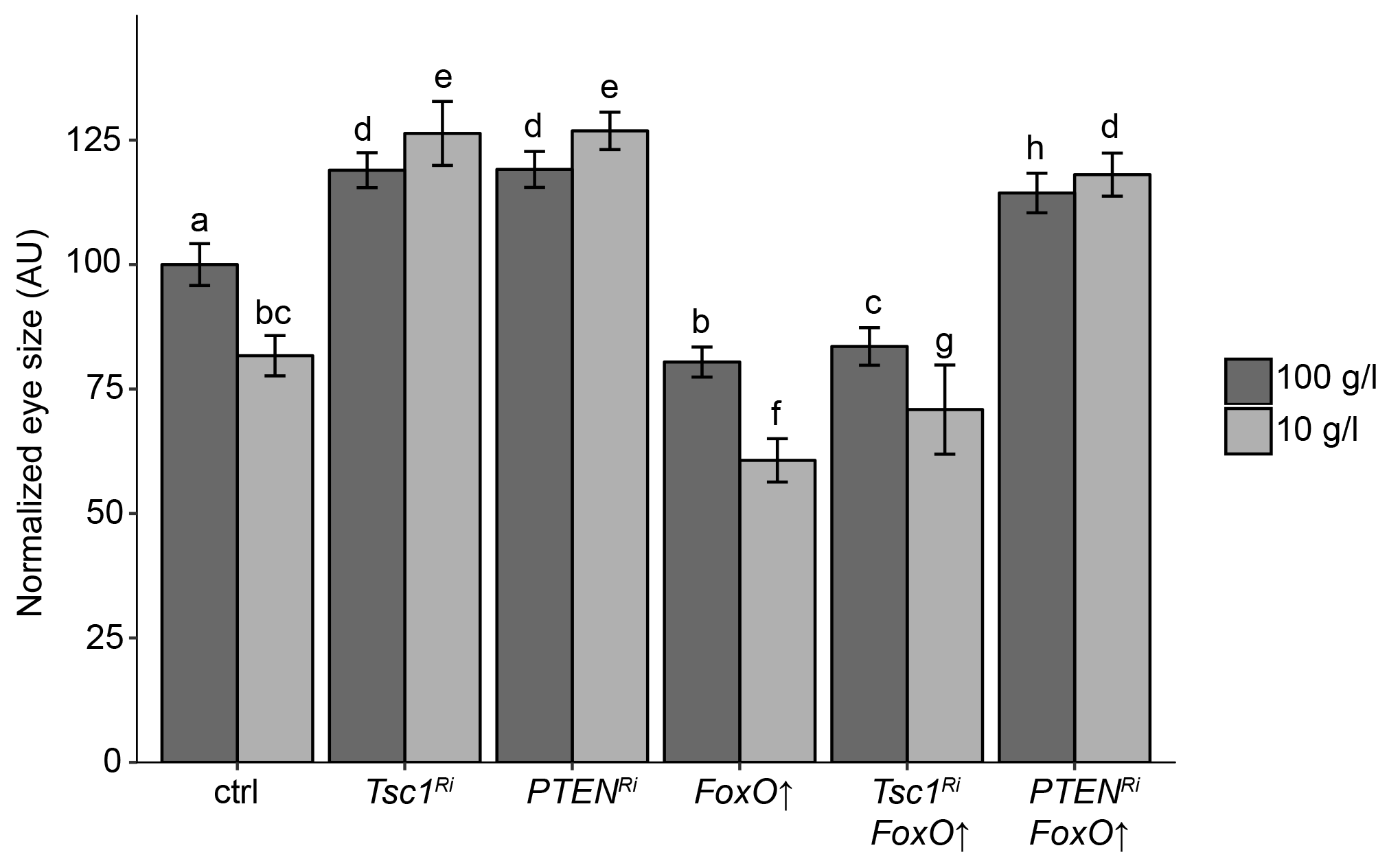

Supplement: S6 Fig — Quantification of eyes shown in Fig 4D. (TIF) [file pgen.1007347.s006.tif]

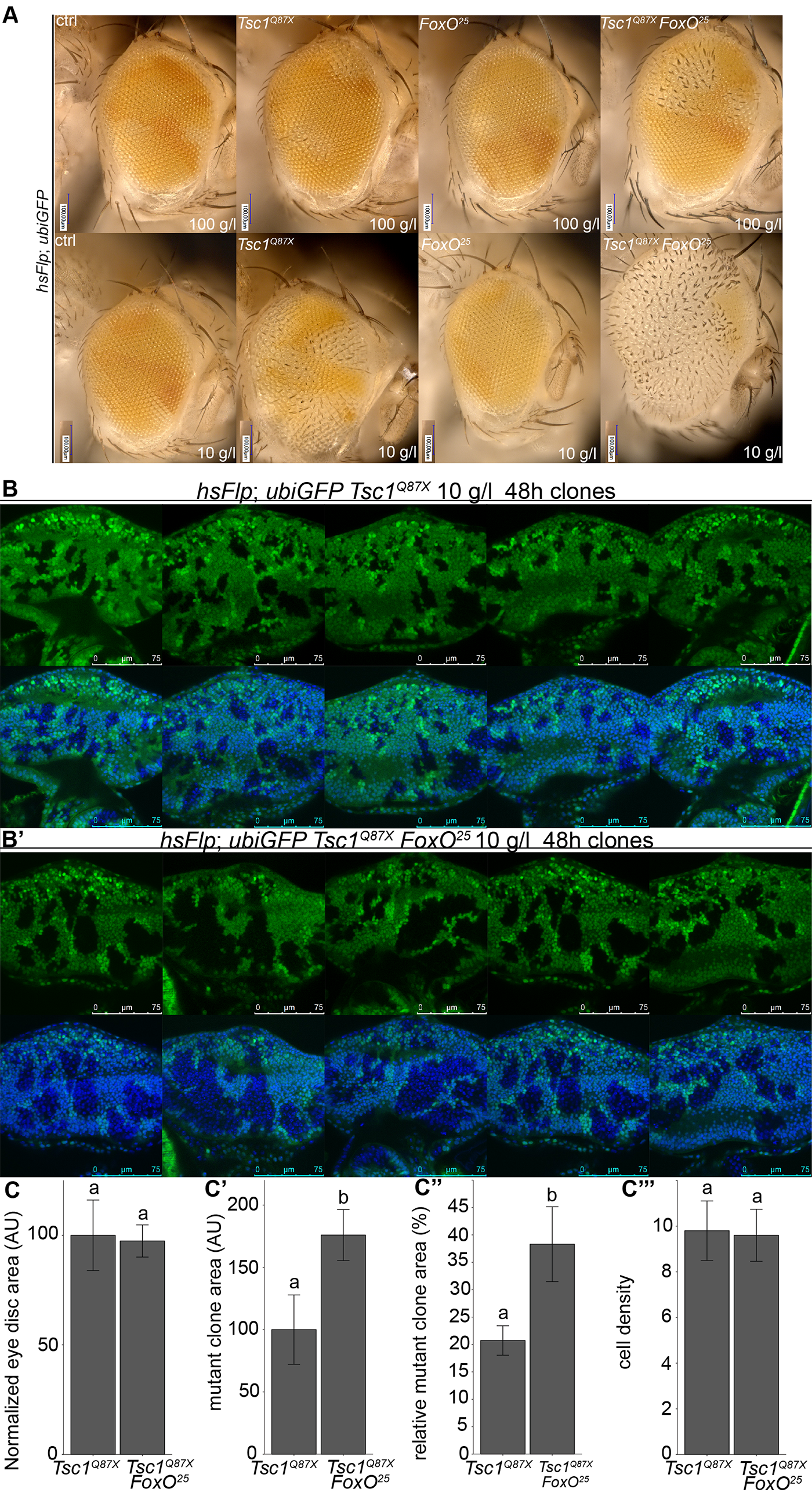

Supplement: S7 Fig — (A) Eyes with hsFlp control, Tsc1, FoxO and Tsc1 FoxO mutant clones (marked by the absence of pigmentation) of animals reared on normal food and NR. (B, B’) Eye discs with hsFlp Tsc1 and Tsc1 FoxO clones (marked by the absence of GFP) dissected from larvae reared on NR 48 h after clone induction (scale bars are 75 μm), and the quantification of (C) eye disc area, (C’) mutant clone area, (C”) mutant clone area relative to the whole disc, and (C‴) mutant cell density (number of nuclei per arbitrary square). (TIF) [file pgen.1007347.s007.tif]

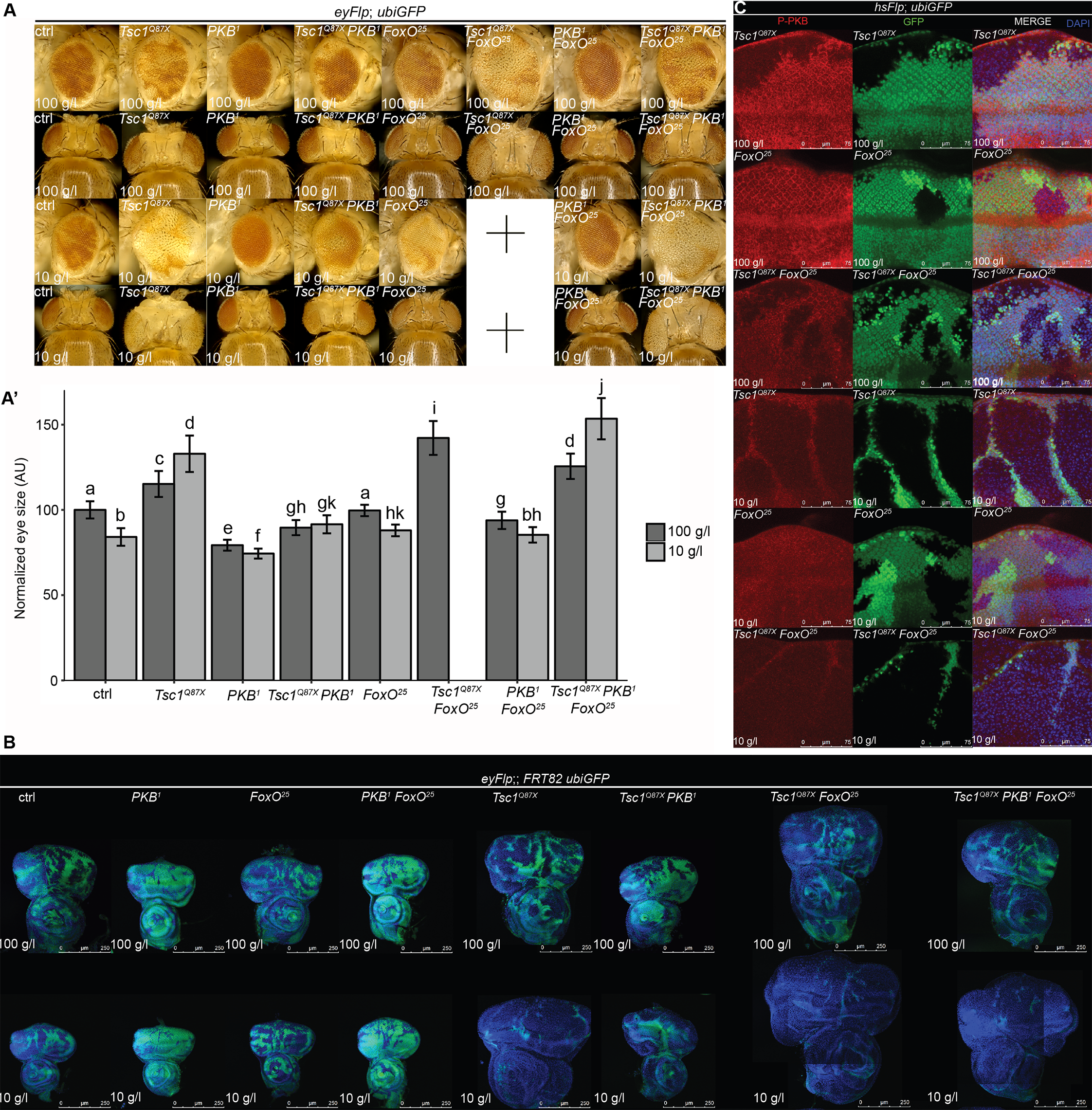

Supplement: S8 Fig — (A) Eyes bearing eyFlp control, Tsc1, PKB or Tsc1 PKB mutant clones (marked by the absence of pigmentation), with or without FoxO mutation, of animals reared on normal food and NR; (A’) quantification of eye size. (B) Eye discs bearing eyFlp control, PKB, FoxO or PKB FoxO mutant clones (marked by the absence of GFP), with or without Tsc1 mutation, dissected from larvae reared on normal food or NR. Scale bars are 250 μm. (C) Phospho-PKB staining (in red) of eyes discs with hsFlp Tsc1, FoxO or Tsc1 FoxO mutant clones (marked by the absence of GFP) dissected from larvae reared on normal food and NR. Scale bars are 75 μm. (TIF) [file pgen.1007347.s008.tif]

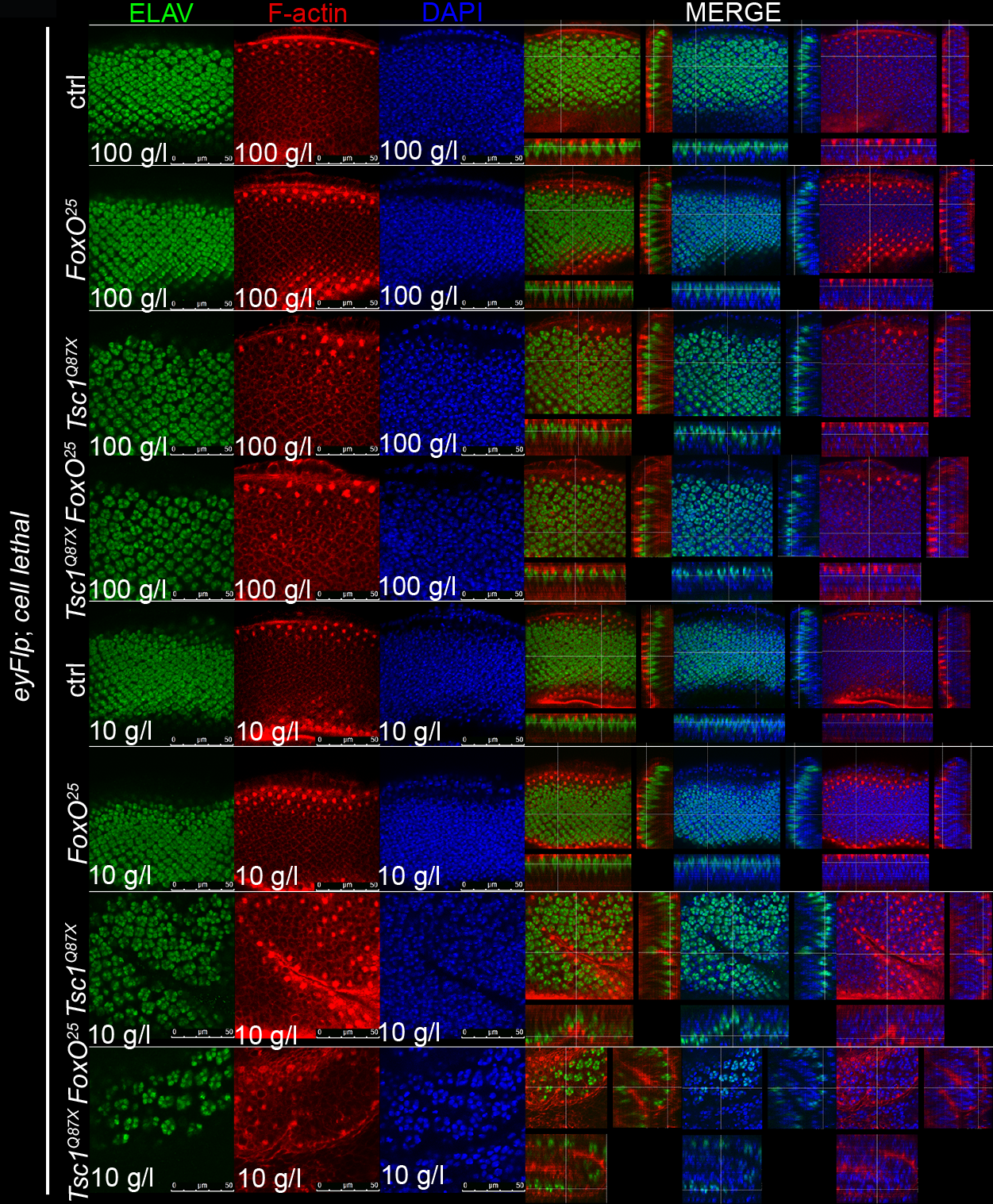

Supplement: S9 Fig — Elav (in green), phalloidin (in red) and DAPI (in blue) stainings in orthogonal sections of eye discs with control, FoxO, Tsc1 and Tsc1 FoxO mutant tissue dissected from larvae reared on normal food and NR. Scale bars are 50 μm. (TIF) [file pgen.1007347.s009.tif]

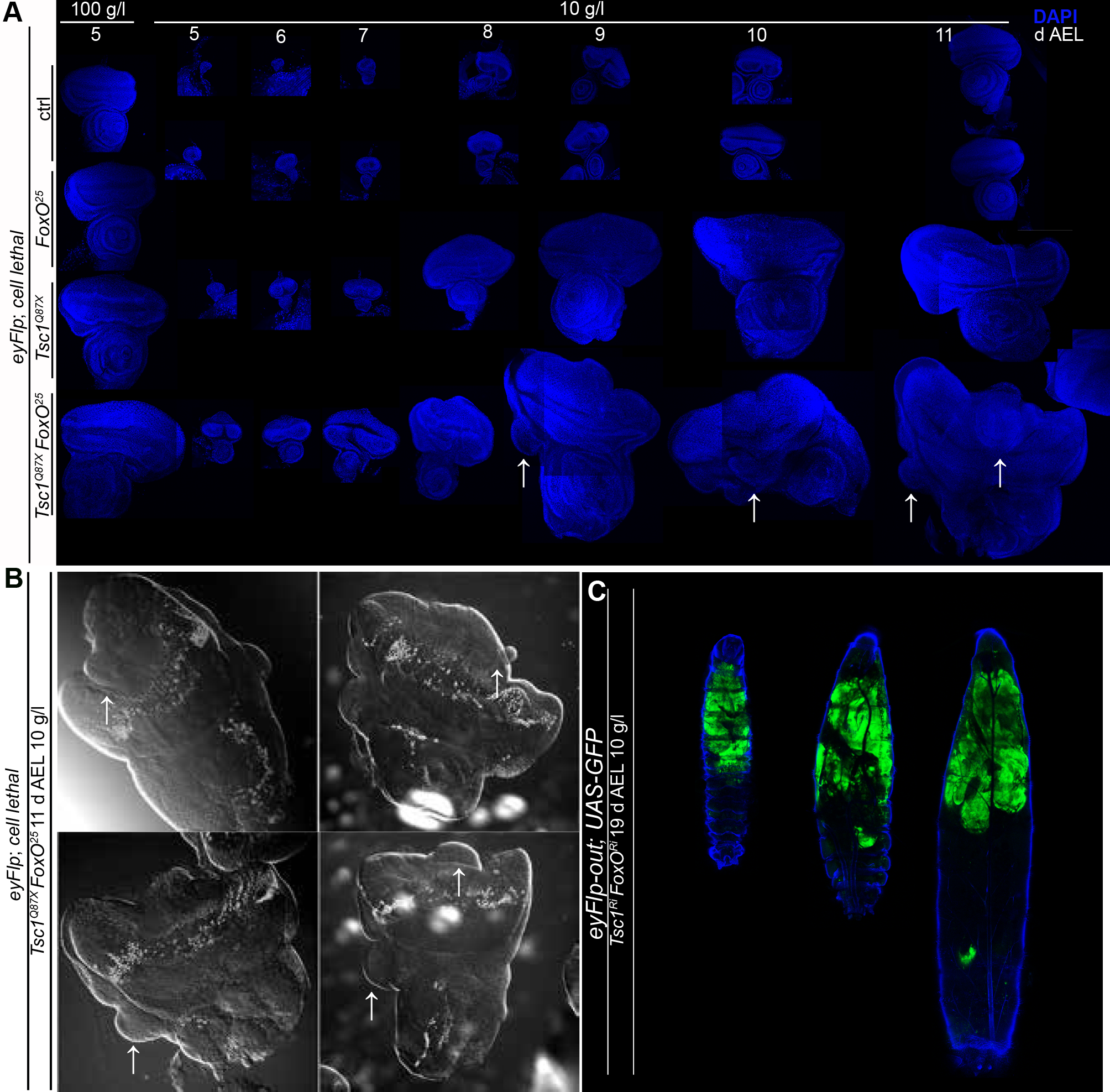

Supplement: S10 Fig — (A) Comparison of development of eye discs with control, FoxO, Tsc1 or Tsc1 FoxO mutant tissue, dissected at indicated time points from larvae reared on normal food and NR. White arrows indicate morphological defects. (B) DIC images of eye discs with Tsc1 FoxO mutant tissue dissected 11 days after egg lay from larvae reared on NR. White arrows indicate morphological defects. (C) Comparison of size of the eye discs with Tsc1 FoxO knockdown tissue (marked by GFP) to larval size for various larvae (n = 46) reared on NR and imaged 19 days AEL. (TIF) [file pgen.1007347.s010.tif]
