# Supplementary material for: FoxO restricts growth and differentiation of cells with elevated TORC1 activity under nutrient restriction
Source: PLoS Genet. 2018 Apr 20;14(4):e1007347. doi: 10.1371/journal.pgen.1007347 (PMC5931687; doi:10.1371/journal.pgen.1007347)
Supplement: S1 Genotypes — (PDF) [file pgen.1007347.s016.pdf]

S1 Genotypes

Fig 1

(A,B) *y w hsFlp/y w;; FRT82 ubiGFP/FRT82 iso*, *y w hsFlp/y w;; FRT82 ubiGFP/ FRT82 Tsc1*^{Q87X}

(C) *y w eyFlp/y w;; FRT82 ubiGFP/FRT82 iso*, *y w eyFlp/y w;; FRT82 ubiGFP/FRT82 Tsc1*^{Q87X}*, (D) y w eyFlp/y w;; FRT82 w*$^{+}$ *cl/FRT82 iso*, *y w eyFlp/y w;; FRT82 w*$^{+}$ *cl/FRT82 Tsc1*^{Q87X}

Fig 2

(A) *y w eyFlp/y w;; FRT82 w*$^{+}$ *cl/FRT82 iso*, *y w eyFlp/y w;; FRT82 w*$^{+}$ *cl/FRT82 Tsc1*^{Q87X}

Fig 3

(A) *y w eyFlp/y w;; FRT82 w*$^{+}$ *cl/FRT82 iso, y w eyFlp/y w;; FRT82 w*$^{+}$ *cl/FRT82 Tsc1*^{Q87X}

(B) *y w eyFlp/y w;; FRT82 ubiGFP/FRT82 iso, y w eyFlp/y w;; FRT82 ubiGFP/FRT82 Rheb*^{2G5}*, y w eyFlp/y w;; FRT82 ubiGFP/FRT82 Tsc1*^{Q87X}*, y w eyFlp/y w;; FRT82 ubiGFP/FRT82 Rheb*^{2G5} *Tsc1*^{Q87X}

(C) *y w hsFlp UAS-GFP/ y w;; tubGal4 FRT82 tubGal80/FRT82 iso, y w hsFlp UAS-GFP/ y w;; tubGal4 FRT82 tubGal80/FRT82 Tsc1*^{Q87X}*, y w hsFlp UAS-GFP/ y w; UAS-Raptor*^{Ri}*/+; tubGal4 FRT82 tubGal80/FRT82 iso, y w hsFlp UAS-GFP/ y w; UAS-Raptor*^{Ri}*/+; tubGal4 FRT82 tubGal80/FRT82 Tsc1*^{Q87X}*, y w hsFlp UAS-GFP/UAS-S6K*^{Ri}*;; tubGal4 FRT82 tubGal80/ FRT82 iso, y w hsFlp UAS-GFP/UAS-S6K*^{Ri}*;; tubGal4 FRT82 tubGal80/ FRT82 Tsc1*^{Q87X}

Fig 4

(A) *y w hsFlp/y w;; FRT82 ubiGFP/FRT82 Tsc1*^{Q87X}*, y w hsFlp/y w; FRT40 ubiGFP/FRT40 PTEN*^{117}

(B) *y w eyFlp/y w;; FRT82 w*$^{+}$ *cl/FRT82 iso, y w eyFlp/y w;; FRT82 w*$^{+}$ *cl/FRT82 Tsc1*^{Q87X}

(C) y *w hsFlp/y w;; FRT82 ubiGFP/FRT82 iso, y w hsFlp/y w;; FRT82 ubiGFP/FRT82 Tsc1*^{Q87X}*, y w hsFlp/y w; FRT40 ubiGFP/FRT40 PTEN*^{117}

(D) *y w eyFlp Act>CD2>Gal4/y w;; UAS-lacZ /+, y w eyFlp Act>CD2>Gal4/y w;; UAS-Tsc1*^{Ri}*/+, y w eyFlp Act>CD2>Gal4/y w; UAS-PTEN*^{Ri}*/+, y w eyFlp Act>CD2>Gal4/y w; UAS-cherry-dFoxO/+, y w eyFlp Act>CD2>Gal4/y w; UAS-cherry-dFoxO/+; UAS-Tsc1*^{Ri}*/+, y w eyFlp Act>CD2>Gal4/y w; UAS-cherry-dFoxO/UAS-PTEN*^{Ri}

Fig 5

(A) y *w eyFlp/y w;; FRT82 ubiGFP/FRT82 iso, y w eyFlp/y w;; FRT82 ubiGFP/FRT82 Tsc1*^{Q87X}*, y w eyFlp/y w; FRT40 ubiGFP/FRT40 PTEN*^{117}*, y w eyFlp/y w;; FRT82 ubiGFP/FRT82 FoxO*^{25}*, y w eyFlp/y w;; FRT82 ubiGFP/FRT82 Tsc1*^{Q87X} *FoxO*^{25}*, y w eyFlp/y w; FRT40 ubiGFP/FRT40 PTEN*^{117}*; FRT82 ubiGFP/FRT82 FoxO*^{25}

(B) *y w eyFlp/y w;; FRT82 w*$^{+}$ *cl/FRT82 iso, y w eyFlp/y w;; FRT82 w*$^{+}$ *cl/FRT82 FoxO*^{25}*, y w eyFlp/y w;; FRT82 w*$^{+}$ *cl/FRT82 Tsc1*^{Q87X}*, y w eyFlp/y w;; FRT82 w*$^{+}$ *cl/FRT82 Tsc1*^{Q87X} *FoxO*^{25}

(C) *y w hsFlp/y w;; FRT82 ubiGFP/FRT82 iso, y w hsFlp/y w;; FRT82 ubiGFP/FRT82 FoxO*^{25}*, y w hsFlp/y w;; FRT82 ubiGFP/FRT82 Tsc1*^{Q87X}*, y w hsFlp/y w;; FRT82 ubiGFP/FRT82 Tsc1*^{Q87X} *FoxO*^{25}

Fig 6

(A) *y w hsFlp/y w;; FRT82 ubiGFP/FRT82 FoxO*$^{\Delta94}$*, y w hsFlp/y w;; FRT82 ubiGFP/FRT82 Tsc1*^{Q87X}*, y w hsFlp/y w;; FRT82 ubiGFP/FRT82 Tsc1*^{Q87X} *FoxO*$^{\Delta94}$

(B) *y w hsFlp UAS-GFP/y w;; tubGal4 FRT82 tubGal80/FRT82 Tsc1*^{Q87X}*, y w hsFlp UAS-GFP/y w; UAS-p35/+; tubGal4 FRT82 tubGal80/FRT82 Tsc1*^{Q87X}*, y w hsFlp UAS-GFP/y w;; tubGal4 FRT82 tubGal80/FRT82 Tsc1*^{Q87X} *FoxO*^{25}*, y w hsFlp UAS-GFP/y w; UAS-p35/+; tubGal4 FRT82 tubGal80/FRT82 Tsc1*^{Q87X} *FoxO*^{25}

Fig 7

(A,A') *y w eyFlp/y w;; FRT82 w*$^{+}$ *cl/FRT82 iso, y w eyFlp/y w;; FRT82 w*$^{+}$ *cl/FRT82 FoxO*^{25}*, y w eyFlp/y w;; FRT82 w*$^{+}$ *cl/FRT82 Tsc1*^{Q87X}*, y w eyFlp/y w;; FRT82 w*$^{+}$ *cl/FRT82 Tsc1*^{Q87X} *FoxO*^{25}

(B) *y w hsFlp/y w;; FRT82 ubiGFP/FRT82 iso, y w hsFlp/y w;; FRT82 ubiGFP/FRT82 FoxO*^{25}*, y w hsFlp/y w;; FRT82 ubiGFP/FRT82 Tsc1*^{Q87X}*, y w hsFlp/y w;; FRT82 ubiGFP/FRT82 Tsc1*^{Q87X} *FoxO*^{25}

Fig 8

(A-D'') *y w eyFlp Act>CD2>Gal4/y w; UAS-FoxO*^{Ri}*/+; UAS-Tsc1*^{Ri}*/+*

(E-H) *y w eyFlp Act>CD2>Gal4/y w; UAS-CG33920*^{Ri}*/+, y w eyFlp Act>CD2>Gal4/y w; UAS-FoxO*^{Ri}*/+, y w eyFlp Act>CD2>Gal4/y w;; UAS-Tsc1*^{Ri}*/+, y w eyFlp Act>CD2>Gal4/y w; UAS-FoxO*^{Ri}*/+; UAS-Tsc1*^{Ri}*/+, y w eyFlp Act>CD2>Gal4/y w; UAS-PTEN*^{Ri}*/+, y w eyFlp Act>CD2>Gal4/y w; UAS-PTEN*^{Ri}*/UAS-FoxO*^{Ri}

S1 Fig

(A,A') *y w eyFlp Act>CD2>Gal4/y w;; UAS-lacZ/+; y w eyFlp Act>CD2>Gal4/y w;; UAS-Tsc2*^{Ri}*/+*

S2 Fig

(A, A') *y w hsFlp/y w;; FRT82 ubiGFP/FRT82 Tsc1*^{Q87X}

S3 Fig

(A) *y w hsFlp UAS-GFP/y w;; tubGal4 FRT82 tubGal80/FRT82 Tsc1*^{Q87X}*, y w hsFlp UAS-GFP/y w; UAS-p35/+; tubGal4 FRT82 tubGal80/FRT82 Tsc1*^{Q87X}

(B) *y w hsFlp/y w;; FRT82 ubiGFP/FRT82 Tsc1*^{Q87X}

S4 Fig

(A) *y w hsFlp UAS-GFP/y w;; tubGal4 FRT82 tubGal80/FRT82 iso, y w hsFlp UAS-GFP/y w; UAS-4EBP*^{WT}*/+; tubGal4 FRT82 tubGal80/FRT82 iso, y w hsFlp UAS-GFP/y w; UAS-4EBP*^{AA}*/+; tubGal4 FRT82 tubGal80/FRT82 iso , y w hsFlp UAS-GFP/y w;; tubGal4 FRT82 tubGal80/FRT82 Tsc1*^{Q87X}*, y w hsFlp UAS-GFP/y w; UAS-4EBP*^{WT}*/+; tubGal4 FRT82 tubGal80/FRT82 Tsc1*^{Q87X}*, y w hsFlp UAS-GFP/y w; UAS-4EBP*^{AA}*/+; tubGal4 FRT82 tubGal80/FRT82 Tsc1*^{Q87X}

S5 Fig

(A) *y w hsFlp UAS-GFP/y w; tub-Gal80 FRT40/FRT40 iso; tub-Gal4/EP-Rheb*

(B) *y w eyFlp Act>CD2>Gal4/y w;; EP-Rheb/+*

(C) *y w hsFlp UAS-GFP/y w; tub-Gal80 FRT40/FRT40 iso; tub-Gal4/EP-Rheb*

S7 Fig

(A) *y w hsFlp/y w;; FRT82 ubiGFP/FRT82 iso, y w hsFlp/y w;; FRT82 ubiGFP/FRT82* $Tsc1^{Q87X}$*, y w hsFlp/y w;; FRT82 ubiGFP/FRT82* $FoxO^{25}$*, y w hsFlp/y w;; FRT82 ubiGFP/FRT82* $Tsc1^{Q87X}$ $FoxO^{25}$

(B) *y w hsFlp/y w;; FRT82 ubiGFP/FRT82* $Tsc1^{Q87X}$*, y w hsFlp/y w;; FRT82 ubiGFP/FRT82* $Tsc1^{Q87X}$ $FoxO^{25}$

S8 Fig

(A,B) *y w eyFlp/y w;; FRT82 ubiGFP/FRT82 iso, y w eyFlp/y w;; FRT82 ubiGFP/FRT82* $Tsc1^{Q87X}$*, y w eyFlp/y w;; FRT82 ubiGFP/FRT82* PKB^{1}*, y w eyFlp/y w;; FRT82 ubiGFP/FRT82* $Tsc1^{Q87X}$ PKB^{1}*, y w eyFlp/y w;; FRT82 ubiGFP/FRT82* $FoxO^{25}$*, y w eyFlp/y w;; FRT82 ubiGFP/FRT82* $Tsc1^{Q87X}$ $FoxO^{25}$*, y w eyFlp/y w;; FRT82 ubiGFP/FRT82* PKB^{1} $FoxO^{25}$*, y w eyFlp/y w;; FRT82 ubiGFP/FRT82* $Tsc1^{Q87X}$ PKB^{1} $FoxO^{25}$

(C) *y w hsFlp/y w;; FRT82 ubiGFP/FRT82* $Tsc1^{Q87X}$*, y w hsFlp/y w;; FRT82 ubiGFP/FRT82* $FoxO^{25}$*, y w hsFlp/y w;; FRT82 ubiGFP/FRT82* $Tsc1^{Q87X}$ $FoxO^{25}$

S9 Fig

y w eyFlp/y w;; FRT82 w^{+} *cl/FRT82 iso, y w eyFlp/y w;; FRT82* w^{+} *cl/FRT82* $FoxO^{25}$*, y w eyFlp/y w;; FRT82* w^{+} *cl/FRT82* $Tsc1^{Q87X}$*, y w eyFlp/y w;; FRT82* w^{+} *cl/FRT82* $Tsc1^{Q87X}$ $FoxO^{25}$

S10 Fig

(A) *y w eyFlp/y w;; FRT82* w^{+} *cl/FRT82 iso, y w eyFlp/y w;; FRT82* w^{+} *cl/FRT82* $FoxO^{25}$*, y w eyFlp/y w;; FRT82* w^{+} *cl/FRT82* $Tsc1^{Q87X}$*, y w eyFlp/y w;; FRT82* w^{+}

cl/FRT82 $Tsc1^{Q87X}$ $FoxO^{25}$

(B) *y w eyFlp/y w;; FRT82* w^{+} *cl/FRT82* $Tsc1^{Q87X}$ $FoxO^{25}$

(C) *y w eyFlp Act>CD2>Gal4/y w;* $UAS\text{-}FoxO^{Ri}$*/+; UAS-GFP/*$UAS\text{-}Tsc1^{Ri}$

S1 Video

y w eyFlp Act>CD2>Gal4/y w; $UAS\text{-}CG33920^{Ri}$*/+, y w eyFlp Act>CD2>Gal4/y w;;* $UAS\text{-}Tsc1^{Ri}$*/+*

S2 Video

y w eyFlp Act>CD2>Gal4/y w; $UAS\text{-}CG33920^{Ri}$*/+, y w eyFlp Act>CD2>Gal4/y w;* $UAS\text{-}FoxO^{Ri}$*/+;* $UAS\text{-}Tsc1^{Ri}$*/+*

S3 Video

y w eyFlp Act>CD2>Gal4/y w; $UAS\text{-}CG33920^{Ri}$*/+, y w eyFlp Act>CD2>Gal4/y w;* $UAS\text{-}PTEN^{Ri}$*/+*

S4 Video

y w eyFlp Act>CD2>Gal4/y w; $UAS\text{-}CG33920^{Ri}$*/+, y w eyFlp Act>CD2>Gal4/y w;* $UAS\text{-}PTEN^{Ri}$*/*$UAS\text{-}FoxO^{Ri}$
